# Evaluating Large Language Models for Preoperative Patient Education in Superior Capsular Reconstruction: Comparative Study of Claude, GPT, and Gemini

**DOI:** 10.2196/70047

**Published:** 2025-06-12

**Authors:** Yukang Liu, Hua Li, Jianfeng Ouyang, Zhaowen Xue, Min Wang, Hebei He, Bin Song, Xiaofei Zheng, Wenyi Gan

**Affiliations:** 1 The Second School of Clinical Medicine, Southern Medical University, Guangzhou, China; 2 Department of Orthopedics, Beijing Jishuitan Hospital, Beijing, China; 3 Zhuhai People's Hospital (The Affiliated Hospital of Beijing Institute of Technology, Zhuhai Clinical Medical College of Jinan University), 79 Kangning Road, Xiangzhou District, Zhuhai, Guangdong, 519000, China, 86 13076855735; 4 Department of Sports Medicine, The First Affiliated Hospital of Jinan University, Guangzhou, China; 5 Department of Orthopaedics, Guangzhou Red Cross Hospital of Jinan University, Guangzhou, China; 6 Department of Joint Surgery and Sports Medicine, The Sixth Affiliated Hospital of Sun Yat-sen University, Guangzhou, China

**Keywords:** superior capsular reconstruction, massive rotator cuff tear, large language models, preoperative patient education, informed consent process

## Abstract

**Background:**

Large language models (LLMs) are revolutionizing natural language processing, increasingly applied in clinical settings to enhance preoperative patient education.

**Objective:**

This study aimed to evaluate the effectiveness and applicability of various LLMs in preoperative patient education by analyzing their responses to superior capsular reconstruction (SCR)–related inquiries.

**Methods:**

In total, 10 sports medicine clinical experts formulated 11 SCR issues and developed preoperative patient education strategies during a webinar, inputting 12 text commands into Claude-3-Opus (Anthropic), GPT-4-Turbo (OpenAI), and Gemini-1.5-Pro (Google DeepMind). A total of 3 experts assessed the language models’ responses for correctness, completeness, logic, potential harm, and overall satisfaction, while preoperative education documents were evaluated using DISCERN questionnaire and Patient Education Materials Assessment Tool instruments, and reviewed by 5 postoperative patients for readability and educational value; readability of all responses was also analyzed using the cntext package and py-readability-metrics.

**Results:**

Between July 1 and August 17, 2024, sports medicine experts and patients evaluated 33 responses and 3 preoperative patient education documents generated by 3 language models regarding SCR surgery. For the 11 query responses, clinicians rated Gemini significantly higher than Claude in all categories (*P*<.05) and higher than GPT in completeness, risk avoidance, and overall rating (*P*<.05). For the 3 educational documents, Gemini’s Patient Education Materials Assessment Tool score significantly exceeded Claude’s (*P*=.03), and patients rated Gemini’s materials superior in all aspects, with significant differences in educational quality versus Claude (*P*=.02) and overall satisfaction versus both Claude (*P*<.01) and GPT (*P*=.01). GPT had significantly higher readability than Claude on 3 R-based metrics (*P*<.01). Interrater agreement was high among clinicians and fair among patients.

**Conclusions:**

Claude-3-Opus, GPT-4-Turbo, and Gemini-1.5-Pro effectively generated readable presurgical education materials but lacked citations and failed to discuss alternative treatments or the risks of forgoing SCR surgery, highlighting the need for expert oversight when using these LLMs in patient education.

## Introduction

Large language models (LLMs) are extensive neural network models based on deep learning [1,2]. These models learn the grammar, semantics, and contextual information of a language by training on vast amounts of textual data, enabling them to perform various natural language processing tasks [1,2]. Due to the powerful text processing, text generation capabilities, and immense knowledge training of LLMs, researchers have begun to continually explore the potential of LLMs in clinical application scenarios, including professional licensing examinations in various countries and regions [3-5], answering public health questions [6,7], analyzing radiological images [8], disease screening [9], disease diagnosis [10], and discipline education [11]. As the versions and functions of LLMs are constantly updated and upgraded, these models have a low usage threshold and are convenient to use. It is particularly important for professionals in various disciplines to assess the accuracy and completeness of LLMs in their respective fields. This assessment not only provides a strong basis for the application of LLMs in various disciplines but also identifies their shortcomings, serving as a warning for nonprofessional users [3,8,10,11].

Superior capsular reconstruction (SCR) was initially proposed by Mihata et al [12] in 2012 as a technique to restore the superior restraint of the humeral head passively, thereby restoring force couples and improving shoulder joint kinematics. Over the past decade, SCR has become one of the commonly used treatment methods for massive and irreparable rotator cuff tears among clinicians [13,14]. However, the surgical techniques for SCR are highly variable [15]. For example, contrary to the results of earlier studies, further research suggests using dermal allograft instead of fascia lata autograft, leading to a current lack of sufficiently effective long-term follow-up data with high levels of evidence [16-18]. Moreover, as SCR is a reconstructive surgery rather than a repair surgery [15], it is challenging to provide patients with a standardized and effective explanation and communication during the preoperative informed consent process. An effective preoperative informed consent process is one of the essential steps in alleviating patients’ perioperative anxiety and improving treatment efficacy [19,20].

Rational and effective preoperative patient education is one of the critical components in developing standardized diagnosis and treatment processes for clinical surgery departments [21]. The main difficulty lies in the professional knowledge gap between medical staff and patients [22]. Previous studies have shown that using multimedia as patient education materials can better help patients understand surgical procedures and alleviate perioperative anxiety [23,24]. However, in most cases, doctors still primarily use verbal responses to address patients’ individualized questions [25]. This might probably because preparing personalized educational materials and providing oral education requires a significant investment of time and effort, leading to high time and economic costs. Furthermore, there is a vast difference in the sources of medical information accessed by doctors and patients [26]. Doctors primarily obtain medical information from clinical guidelines, research literature, and textbooks, while patients often acquire medical information through simple search engines and social media software, which may contain false and overly embellished content [26-28]. Patients often lack the ability to think independently when faced with this information.

With the development of LLMs in recent years, researchers have discovered that the disciplinary knowledge possessed by these LLMs can pass professional examinations in multiple disciplines [3,10,29]. Their powerful text processing capabilities not only allow them to polish complex text content to enhance readability but also enable them to independently generate text content that is more comprehensive and empathetic compared to health care professionals [6,7,30]. The quality of their answers is also significantly better than the search results from search engines [27,28]. Researchers have also pointed out that when using LLMs as patient education assistive tools, the primary task of doctors is to determine the accuracy of the information and make necessary clarifications [5,31]. Furthermore, researchers believe that LLMs can present information in a way that is understandable to most patients, making them a valuable supplement for orthopedic surgeons in obtaining informed consent and shared decision-making [4,5].

This cross-sectional study aims to assess the capability and application potential of different LLMs in preoperative patient education by evaluating the responses of 3 LLMs—GPT-4-Turbo, Claude-3-Opus, and Gemini-1.5-Pro—to SCR-related patient inquiries. In addition, the study will evaluate patient education documents generated by the LLMs for the informed consent process, which will be jointly assessed by health care professionals and patients. We hypothesize that LLMs can generate readable patient education materials for SCR, but the accuracy, completeness, and patient-assessed readability of the content will require expert review before clinical application.

## Methods

### Study Design Overview

**Figure 1.**
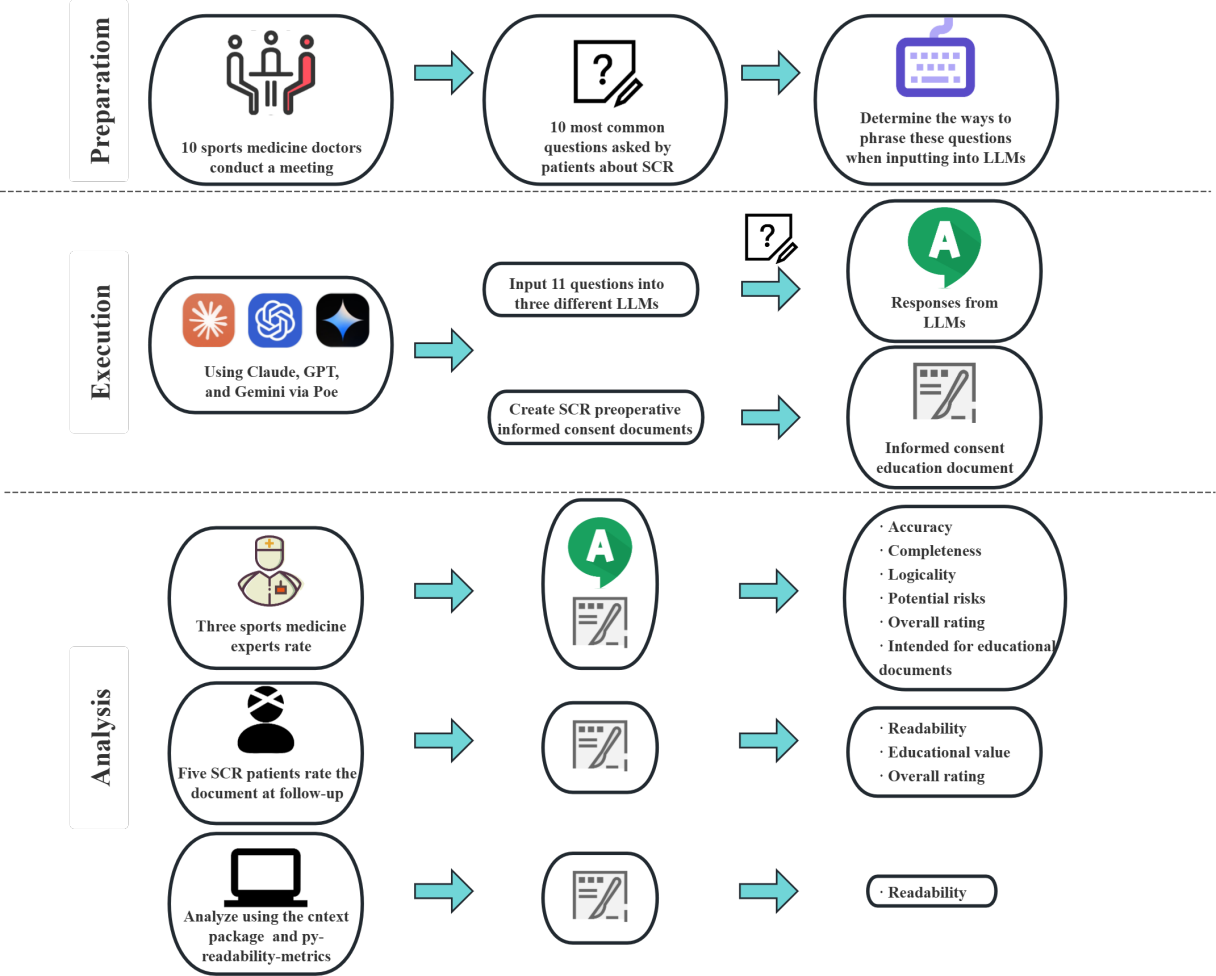
Flow diagram of the study process. LLM: large language model; SCR: superior capsular reconstruction.

This cross-sectional analysis, conducted from July 1 to August 17, 2024, evaluated the quality of responses generated by different LLMs in the context of preoperative patient education for SCR. The study design assessed Claude-3-Opus, GPT-4-Turbo, and Gemini-1.5-Pro (accessed via Poe) on their ability to answer SCR-related patient questions and generate educational materials. The specific study flow is shown in Figure 1. All LLM prompts and responses, as well as expert and patient evaluations, were conducted in Chinese. Screenshots of Poe website operations are available in Mendeley (Mendeley Data, V1), with English translations generated by GPT-4-Turbo (via Poe) in Multimedia Appendix 1.

### Ethical Considerations

This study was approved by the Ethics Committee of our organization and was eligible for exemption from ethical review considering that this cross-sectional study involved no interventions or potential risks to patients.

### Questions and Prompts Development

The research team for this study consists of 12 members, including 10 experienced sports medicine clinicians and 2 doctoral students specializing in LLMs, who collaborated to create patient education materials about SCR. The clinicians include 3 senior-level experts (2 of whom are subject matter experts from external institutions), 2 associate senior-level experts, and 5 intermediate-level experts, with each clinician having at least 5 years of clinical experience.

The 2 doctoral students first collected a total of 100 questions by having each of the 10 clinical experts propose 10 questions daily that patients frequently asked about SCR, covering aspects like etiology, treatment principles, methods, complications, rehabilitation, and hospitalization costs. After removing duplicates and combining some of the questions, they included only the effective questions that all experts agreed were meaningful. This process resulted in the inclusion of 11 questions. Along with these questions, the doctoral students provided instructions (Table 1) requiring LLMs to draft a standardized preoperative informed consent patient education document. After the drafted prompts were reviewed and approved by the aforementioned 10 clinical experts, doctoral students created standardized prompts for each question, consisting of unified “Background+ Question” formats (Table 1). These standardized prompts were then used to generate a comprehensive patient education document addressing most concerns of SCR patients using LLMs.

**Table 1.** Content and strategies for asking questions to large language models.

Subject	Theme	Content
Background	Clinical case	The patient was diagnosed with a massive rotator cuff tear due to supraspinatus muscle injury. The doctor plans to perform a superior capsular reconstruction surgery on the shoulder joint.
Question 1	Muscle injury	The imaging report says that I have a supraspinatus muscle injury. What is the supraspinatus muscle, and what causes this type of injury?
Question 2	Surgical principles and indications	What is the reconstruction of the superior capsule of the shoulder joint, what is the therapeutic principle of the surgery, and what are the indications for the surgery?
Question 3	Graft materials	What are the commonly used graft materials in the reconstruction of the superior capsule of the shoulder joint, and what are the differences between these grafts?
Question 4	Surgical hardware	Besides grafts, does the reconstruction of the superior capsule of the shoulder joint require the use of screws, and do these screws need to be removed in a second surgery?
Question 5	Surgical complications	What are the surgical complications of superior capsule reconstruction of the shoulder joint?
Question 6	Recovery time	How long is the typical recovery time after superior capsule reconstruction surgery of the shoulder joint?
Question 7	Healing issues	What situations can lead to poor healing or failure of the superior capsule reconstruction surgery of the shoulder joint?
Question 8	Autograft risks	In superior capsule reconstruction surgery of the shoulder joint, if an autograft is chosen, what are the impacts and risks to the area from which the autologous tissue is harvested?
Question 9	Surgical costs	What are the chargeable items during the superior capsule reconstruction surgery of the shoulder joint, and what surgical consumables are needed?
Question 10	Graft longevity	If the superior capsule reconstruction surgery of the shoulder joint is successful, how long is the lifespan of the implanted graft, and what are the differences between different types of grafts?
Question 11	Anesthesia and hospitalization	What type of anesthesia is required for superior capsule reconstruction surgery, how long does the surgery take, and how long is the hospital stay required?
Document generation request	Education document	Please generate a comprehensive educational document about superior capsule reconstruction surgery of the shoulder joint. This document is to be provided to patients for reading during the preoperative informed consent process.

### LLM Selection and Prompt Execution

Both ChatGPT 4 and Claude 3 are among the most popular language models today, with Gemini (formerly known as Bard) also gaining significant traction [32]. Studies suggest potential discrepancies in the functionalities of GPT-4 models used on the OpenAI official website [33]. To mitigate potential systematic errors arising from these discrepancies, we access Claude-3-Opus, GPT-4-Turbo, and Gemini-1.5-Pro through the Poe website. Poe, created by Anthropic, is a platform that aggregates multiple AI chatbots, enabling users to engage with different AI assistants within a single interface and compare their responses [34].

To ensure that each interaction is independent and unbiased by previous exchanges, the doctoral students perform a “clear context” operation after each query. This approach ensures that each question and response are treated independently, preventing information carryover from previous interactions, and is informed by other research [7,11]. Since the purpose of our study was to evaluate the ability of pretrained LLMs to handle new tasks, we used LLMs in Zero-shot mode. Before input, the generated content has no specific setting (ie, suppose you are a doctor or speak like a doctor). The input provided to the LLMs follows a “background+ question/request” format (human message) and the output answers (assistant message) were collected then, ensuring clarity and relevance within each independent interaction.

### Evaluation of LLM Response Quality

This study evaluates the quality of patient informed consent documents generated by LLMs from 3 perspectives: physicians’ assessment, patients’ assessment, and readability analysis.

In total, 3 senior doctors evaluated the LLMs’ responses to 11 specific questions related to a specific medical procedure, assessing them for correctness, completeness, logic, and potential harm using a 5-point Likert scale [35]. Physicians also provided an overall satisfaction score using a 10-point Likert scale. In addition, to evaluate the quality of health care information provided by each LLM, 2 validated instruments were also used to assess the generated documents: DISCERN (score ranging from 1=low to 5=high for overall information quality) and the Patient Education Materials Assessment Tool (PEMAT) for printable materials (scores of 0%‐100% for understandability) [6]. The PEMAT assessment tool was able to assess printable and audiovisual understandability, while the DISCERN instrument could review the quality of information for the consumer particularly with a focus on treatment choices in health information.

In total, 5 patients who underwent the specific medical procedure reviewed the LLM-generated patient education documents, rating their readability and educational value on a 5-point Likert scale and overall satisfaction on a 10-point Likert scale. This aimed to assess the documents’ clarity and educational value from nonprofessional readers’ perspectives.

Finally, a readability analysis of all LLMs’ responses was conducted using the cntext package [36] in R (version 4.4.1), examining sentence structure and evaluating readability via 3 indices: readability 1 (average characters per clause), readability 2 (proportion of adverbs and conjunctions), and readability 3, based on the Fog Index and calculated as half the sum of readability 1 and readability 2. Besides, we also applied the “py-readability-metrics” to evaluate the readability, which includes metrics such as the Flesch Reading Ease Score, Flesch-Kincaid Grade Level, and Gunning Fog Index.

### Data Analysis

Statistical analysis used SPSS (version 26.0; IBM Corp) using nonparametric tests due to nonnormally distributed data (Kolmogorov-Smirnov test). Mann-Whitney *U* test compared scoring between groups, with significance at *P*<.05. Interrater reliability, assessed using Fleiss kappa value, was interpreted as follows: poor agreement (<0.01); slight agreement (0.01‐0.20); fair agreement (0.21‐0.40); moderate agreement (0.41‐0.60); substantial agreement (0.61‐0.80); almost perfect agreement (0.81‐1.00) [7]. GraphPad Prism 8 generated bar charts for visualizing results.

## Results

### Overview

Between July 1 and July 14, 2024, we sent invitations to sports medicine experts at various hospitals in the South China region for a webinar held on July 18. During this meeting, we discussed 11 key issues and formulated 12 strategies for sending inquiry requests as part of our study. From July 20 to August 1, 2024, we posed 11 surgery-related questions about SCR and requested the creation of preoperative patient education documents through the Poe website to 3 different LLMs: Claude-3-Opus, GPT-4-Turbo, and Gemini-1.5-Pro. These models collectively produced 33 responses and 3 preoperative patient education documents. From August 10 to August 17, 2024, three experienced sports medicine clinicians, who are not from the same institution, along with 5 patients who had undergone SCR surgery, evaluated the responses and documents provided by the LLMs.

### Evaluations From the Subjective Perspective of Doctors

**Figure 2.**
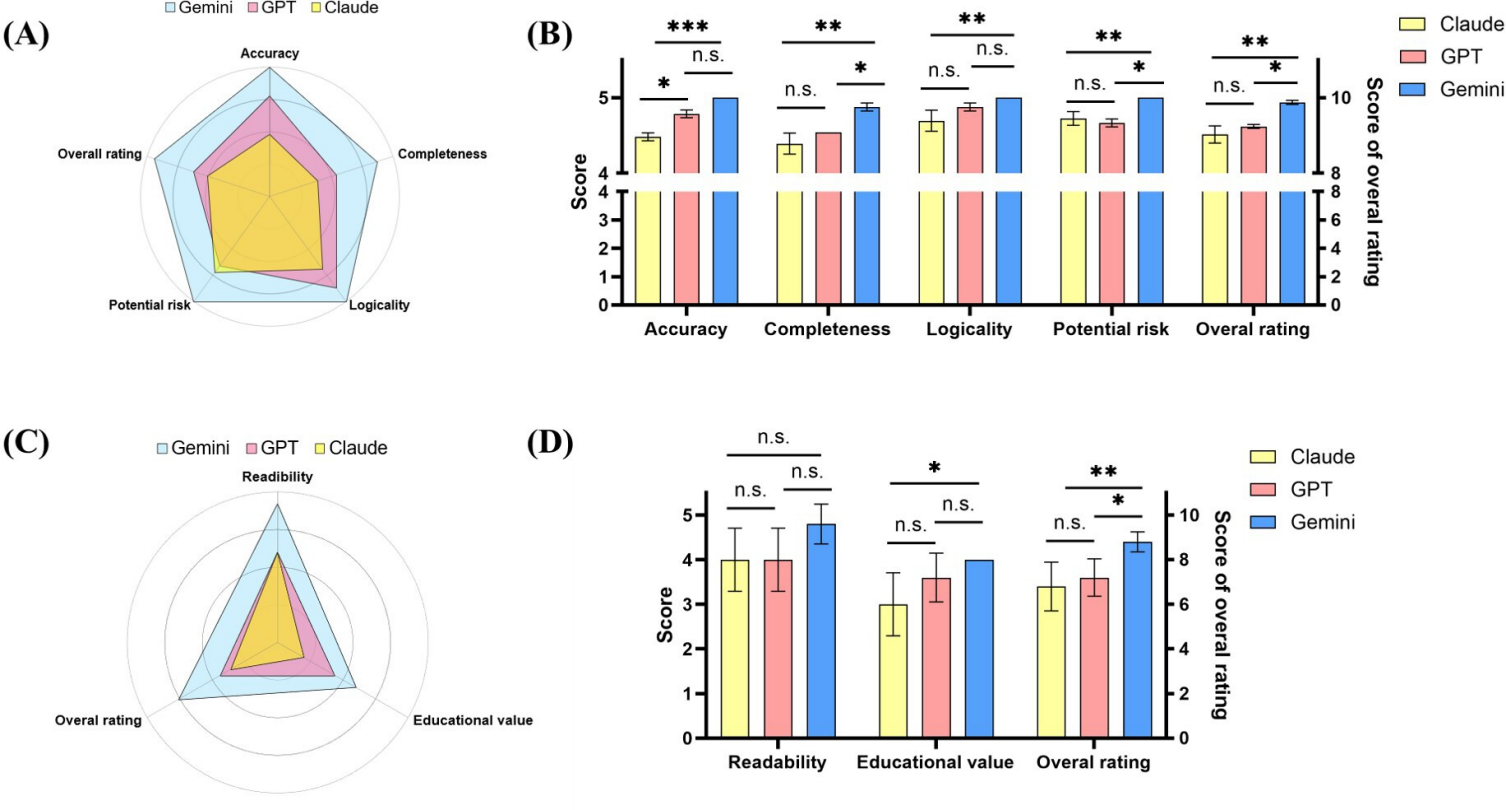
Quality evaluation results from doctors and patients for 11 questions generated by 3 large language models. (**A-B**) Evaluation from the doctor’s perspective; (**C-D**) evaluation from the patient’s perspective. n.s. not significant; *P<.05, **P<.01, ***P<.001.

In total, 3 professional sports medicine doctors first evaluated the responses of 3 different LLMs to 11 inquiries. The evaluations focused on accuracy, completeness, logicality, potential risk, and overall rating. The results showed that Gemini’s responses were significantly superior to Claude’s in all evaluated categories including accuracy (mean 5.00, SD 0.00 vs mean 4.48, SD 0.83; *P*<.001), completeness (mean 4.88, SD 0.33 vs mean 4.39, SD 0.70; *P*=.001), logicality (mean 5.00, SD 0.00 vs mean 4.70, SD 0.59; *P*<.01) potential risk (mean 5.00, SD 0.00 vs mean 4.73, SD 0.57; *P*<.01), and overall rating (mean 9.88, SD 0.42 vs mean 9.03, SD 1.31; *P*=.001; Figures 2A and 2B). Compared to GPT, Gemini’s responses were superior in all categories, with significant differences noted in completeness (mean 4.88, SD 0.33 vs mean 4.55, SD 0.67; *P*=.02), potential risk (mean 5.00, SD 0.00 vs mean 4.67, SD 0.82; *P*=.01), and overall rating (mean 9.88, SD 0.42 vs mean 9.24, SD 1.30; *P*=.01; Figures 2A and 2B. GPT’s responses, when compared to Claude’s, were superior in accuracy (*P*=.03), completeness (*P*=.34), logicality (*P*=.11), and overall rating (*P*=.42); however, Claude was rated higher in potential risk (*P*=.85; Figures 2A and 2B). Of these differences, only the accuracy presented a statistically significant difference (Figures 2A and 2B).

**Figure 3.**
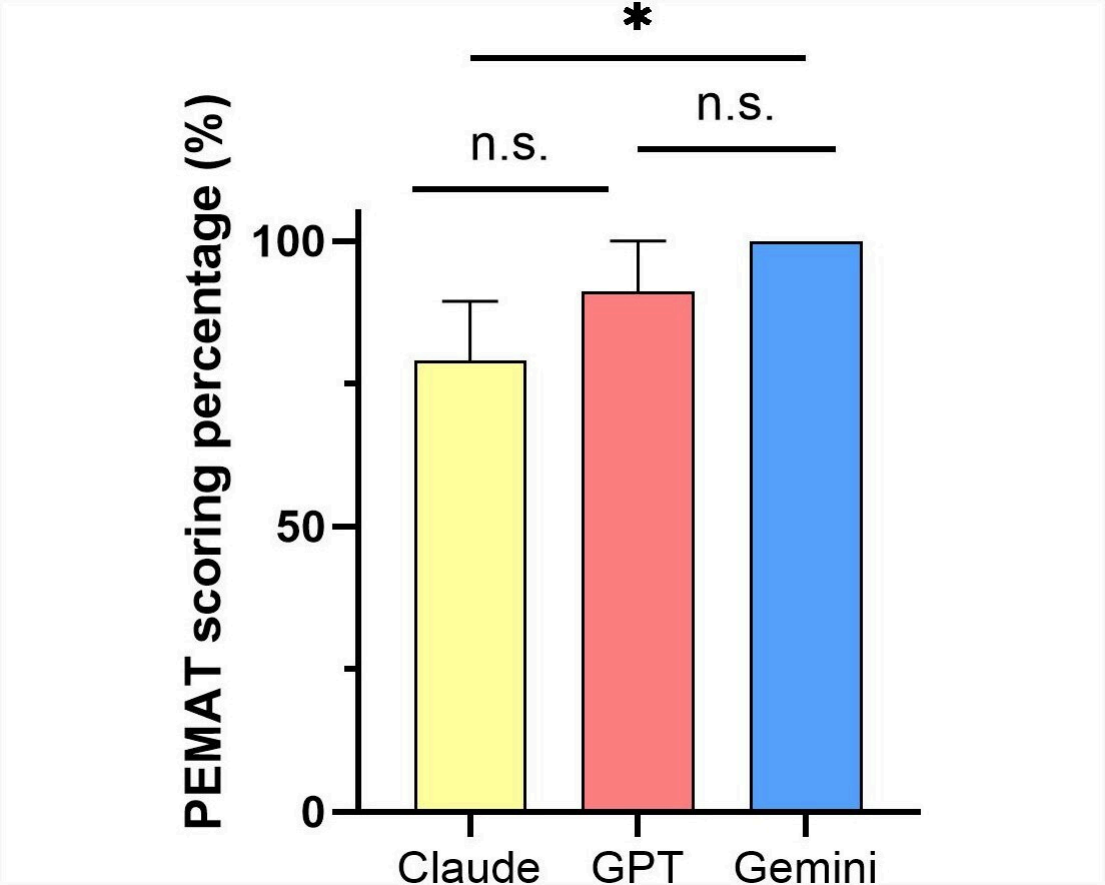
PEMAT scoring percentage for the patient education document generated by three large language models. n.s.: not significant; *P<.05, **P<.01, ***P<.001.

In terms of the PEMAT scores for the preoperative patient education materials generated by each LLM, Gemini scored higher than GPT (mean 1.00, SD 0.00 vs mean 0.91, SD 0.09; *P*=.12), and GPT scored higher than Claude (mean 0.91, SD 0.09 vs mean 0.79, SD 0.10; *P*=.18), with only the difference between Gemini and Claude (mean 1.00, SD 0.00 vs mean 0.79, SD 0.10; *P*=.03) being statistically significant (Figure 3). Regarding the DISCERN scores, Claude achieved the highest overall score, followed by Gemini and then GPT, though these differences were not statistically significant (Table 2). In the item of the DISCERN which represents overall satisfaction (the 16th question presented in Table 2), Gemini scored the highest, while GPT and Claude scored the same, with no statistical significance in the differences. The consistency among the 3 evaluators was high, with no instances of “Poor agreement” or “Slight agreement” in their assessments (Multimedia Appendix 2).

**Table 2.** Quality grades for section 2 of the DISCERN Tool.

Section 2. How good is the quality of information on treatment choices ?	Claude-3-Opus, Median (IQR)	GPT-4-Turbo, Median (IQR)	Gemini-1.5-Pro, Median (IQR)	Claude versus GPT, *P* value	Claude versus Gemini, *P* value	GPT versus Gemini, *P* value
Does it describe how each treatment works?	4 (3-4)	4 (3-4)	5 (4-5)	—^a^	.09	.09
Does it describe the benefits of each treatment?	4 (3-5)	4 (3-4)	1 (1-1)	.64	.04	.03
Does it describe the risks of each treatment?	4 (3-4)	3 (2-3)	5 (4-5)	.09	.09	.04
Does it describe what would happen if no treatment is used?	1 (1-1)	1 (1-1)	1 (1-1)	—	—	—
Does it describe how the treatment choices affect overall quality of life?	1 (1-1)	1 (1-1)	1 (1-1)	—	—	—
Is it clear that there may be more than one possible treatment choice?	1 (1-1)	1 (1-1)	1 (1-1)	—	—	—
Does it provide support for shared decision-making?	3 (3-4)	3 (2-3)	3 (2-3)	.32	.20	—
Based on the answers to all of the above questions, rate the overall quality of the publication as a source of information about treatment choices.	3 (3-4)	3 (3-4)	4 (3-4)	—	.46	.46

a Not applicable.

### Evaluations From the Subjective Perspective of Patients

In the ratings provided by 5 follow-up patients for the preoperative patient education materials generated by the LLMs, Gemini scored higher than GPT and Claude across all parameters, including readability, educational quality, and overall rating (Figures 2C and 2D). Among these, the difference in educational quality between Gemini and Claude (mean 4.00, SD 0.00 vs mean 3.60, SD 0.55; *P*=.02) was statistically significant (Figures 2C and 2D). Furthermore, Gemini’s advantage in overall satisfaction when compared to both Claude (mean 8.80, SD 0.45 vs mean 6.80, SD 1.10; *P*<.01) and GPT (mean 8.80, SD 0.45 vs mean 7.20, SD 0.84; *P*=.01) also showed statistical significance (Figures 2C and 2D). The consistency of all ratings given by the 5 follow-up patients was evaluated as “Fair agreement” (Multimedia Appendix 2).

### Objective Evaluations of Readability

**Figure 4.**
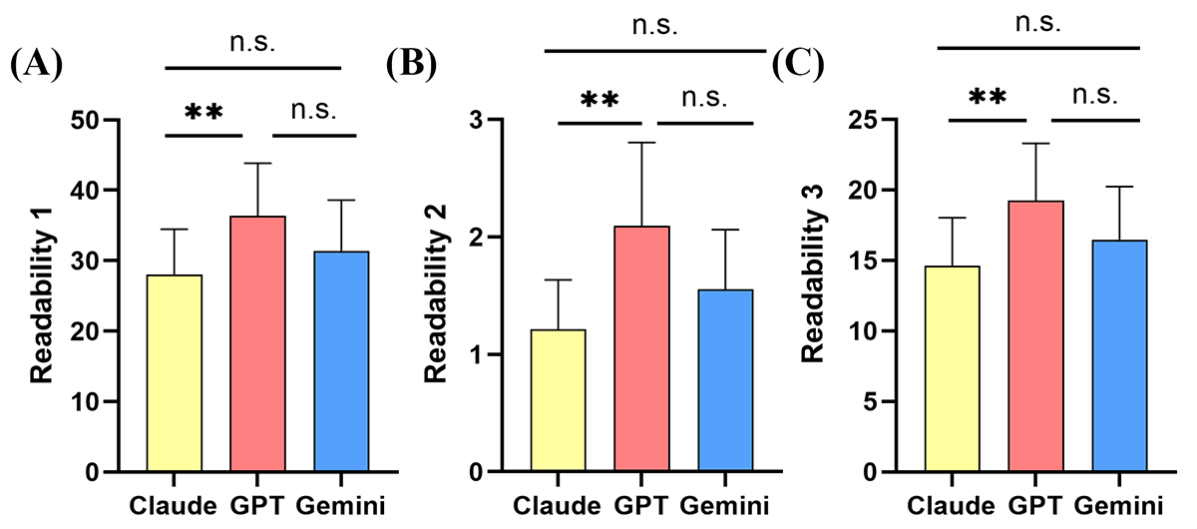
Comparison of the results of text readability analysis from three analytical perspectives using the cntext package in R software. n.s.: not significant; *P<.05, **P<.01, ***P<.001.

Based on the analysis methods of the context package, readability is assessed from 3 perspectives, namely readability 1, readability 2, and readability 3. Under these assessments, GPT’s readability is higher than that of Gemini (readability 1: mean 36.38, SD 7.47 vs mean 31.39, SD 7.20, *P*=.18; readability 2: mean 2.09, SD 0.71 vs mean 1.55, SD 0.51, *P*=.09; readability 3: mean 19.24, SD 4.07 vs mean 16.47, SD 3.77, *P*=.17) and Claude (readability 1: mean 36.38, SD 7.47 vs mean 28.05, SD 6.43, *P*<.01; readability 2: mean 2.09, SD 0.71 vs mean 1.21, SD 0.42, *P<*.01; readability 3: mean 19.24, SD 4.07 vs mean 14.63, SD 3.40, *P*<.01), with the difference between GPT and Claude being statistically significant (Figure 4). Although Gemini’s readability is higher than Claude’s, the difference is not statistically significant (Figure 4). However, when readability was assessed using py-readability metrics, there was no statistical difference between the 3 LLM models (Multimedia Appendix 3).

## Discussion

### Principal Findings

The main findings of our study are as follows: (1) the three LLMs (Claude-3-Opus, GPT-4-Turbo, and Gemini-1.5-Pro) demonstrated good overall potential for application in patient education for SCR surgery. They were able to generate answers to 11 SCR-related questions and create standardized preoperative informed consent patient education documents. (2) In the subjective evaluations by professional sports medicine clinicians and patients who had undergone SCR surgery, Gemini slightly outperformed GPT and Claude in multiple dimensions, including accuracy, completeness, logic, potential risks, and overall satisfaction. (3) In this study, the 3 LLMs did not proactively provide evidence sources when answering questions and generating patient education documents. If LLMs are to be used to assist with patient education in clinical applications, it may be necessary to specifically require LLMs to cite information sources to enable doctors and patients to judge the authority and reliability of the content. (4) Although Gemini performed best in the ratings for SCR patient education-related tasks, considering the complexity and potential risks of LLMs in medical applications, clinicians still need to carefully review and make necessary corrections to the content generated by LLMs to ensure the professionalism and reasonableness of patient education materials. LLMs should be positioned as assistive tools rather than decision-making entities in clinical applications.

LLMs have proven to be reliable sources of information for orthopedic surgery-related questions, creating patient education documents that enhance the understanding of diagnostic and therapeutic processes for nonprofessionals and improve the readability of educational materials [28,37,38]. However, evaluating the quality of responses from LLMs is not straightforward. Researchers assessed ChatGPT 3.5’s medical knowledge by using clinical standards and licensing examination questions to evaluate its theoretical understanding and practical application [39]. With the advent of ChatGPT 4.0 and the iterative upgrades of various LLMs from different companies, there has been a growing recognition and exploration of the expanded pretraining data and enhanced text processing capabilities of the latest LLM versions in different clinical scenarios [40,41]. Scholars have realized that the quality of LLM responses is influenced by multiple factors, including the amount of information in the query [42], the questioning strategy [43], and many unpredictable elements [44]. These unpredictable elements are evident when, under controlled conditions with all variables constant, the same question yields different answers and shows varying styles of text presentation. Consequently, while researchers have acknowledged the capabilities of LLMs in diagnosing, treating, and creating educational documents across disciplines, they continue to reject the idea of LLMs performing independent medical actions, affirming their role solely as an auxiliary tool in the hands of professionals [45,46].

This study aims to assess the feasibility of using three popular LLMs as auxiliary tools for sports medicine physicians during the informed consent process for patients undergoing SCR. In this study, physicians use LLMs primarily to assess the accuracy and comprehensiveness of the information and to clarify content. Unlike previous studies that evaluated answer readability solely through software analysis of word and sentence structure [4,6,47], this study also included follow-up visits with SCR patients post surgery, where patients subjectively assessed the readability and educational significance of the information. Patient ratings primarily focused on the presurgical educational materials generated by LLMs, excluding the evaluation of 11 specific questions, as the answers to these questions required physician assessment of accuracy and comprehensiveness and clarification before clinical use. Without this step by physicians, patients, who are not medical professionals, might not be able to accurately assess the details of the questions. Although all 3 models performed satisfactorily in evaluating “potential risks,” this does not imply that patients can rely on LLMs as their sole source of medical advice. We believe that the SCR medical decision-making process, which does not involve extensive use of medications and auxiliary treatments pre- and post-surgery and follows a “surgery-rehabilitation” model, does not necessitate the phase-wise, continuous assessments and patient education required for conditions like cancer.

Despite the potential benefits of using LLMs in patient education, several ethical and privacy issues need to be addressed before their widespread application. The accuracy and reliability of the information generated by LLMs are critical, especially in sensitive medical contexts. To enhance their accuracy, strategies such as retrieving pertinent information from credible, external data sources before generating text can be incorporated into subsequent versions of LLMs. And patient privacy is a fundamental concern when using LLMs in medical settings. LLMs may require access to patient data to generate personalized and relevant information. However, this access must be strictly regulated to prevent unauthorized use or disclosure of sensitive patient information.

In addition, our “Prompt Execution” phase revealed that without background information, LLMs occasionally misidentify SCR as a supraspinatus repair surgery under patch bridging, leading to content generation biases. We consider such biases to be system errors caused by human operational mistakes, which can be avoided by adjusting prompt strategies under the guidance of subject matter experts. Therefore, using LLMs for specialist information retrieval is not without its challenges, and we believe that merely relying on LLM-generated disclaimers like “I am not a medical professional; if you feel unwell, please seek medical attention immediately” at the end of responses is insufficient [28]. The mitigation of these errors can be facilitated through the use of techniques such as fine-tuning and retrieval-augmented generation. Fine-tuning entails training the LLM on a smaller, highly specialized dataset that has been meticulously curated to capture the intricate details of the medical domain and retrieval-augmented generation can address issues of hallucinations by first retrieving pertinent information from credible, external data sources before generating text. Incorporating these strategies into subsequent versions of LLMs has the potential to enhance their accuracy and reliability, particularly in sensitive applications such as patient education. A thorough examination would offer valuable insights into refining these models to deliver precise and trustworthy information within medical contexts.

Our study meanwhile discovers critical gaps in LLMs are used in medical settings, particularly in presurgical patient education. LLMs often do not provide sources for their information, and their responses can include inaccuracies or fabricated sources, known as “hallucinations” [48]. This issue is exacerbated when users do not specifically ask for sources, leading LLMs to sometimes provide outdated or irrelevant information [48,49]. Furthermore, the LLMs in the study failed to discuss alternative treatments, benefits, and risks associated with not undergoing specific surgeries like SCR. This omission is significant as discussing these elements is essential for informed medical decision-making and respects patient rights to understand all available options. Given these limitations, LLMs should not independently manage diagnosis or patient education. Instead, they should serve as supplementary tools, aiding health care professionals who can provide the necessary context, accuracy, and depth in patient interactions. This approach ensures that patient education remains thorough, accurate, and ethically conducted, aligning with medical standards and patient rights. This challenge can be tackled through the application of more advanced prompt engineering methodologies, the integration of contextual reasoning capabilities, and the implementation of step-by-step guidance mechanisms. By engaging in multiple iterative interactions with the model, it becomes possible to refine its responses and produce more comprehensive information, encompassing alternative treatment options, based on the specific inputs provided by the user. Such an approach would empower the LLM to deliver content that is more personalized, well-informed, and balanced. Moreover, the development of LLM-Agents offers a compelling solution to the limitations of LLMs in sensitive domains like medical decision-making. By integrating planning, memory, tool use, and agent or brain components, these agents can enhance their ability to provide accurate, verified information. This not only supports human expertise but also ensures that the information presented is transparent and evidence-backed. As research continues, the full potential of integrating citation capabilities within LLM-Agents should be explored to further improve their reliability and trustworthiness in high-stakes contexts.

With the evolution of internet technology, we have witnessed a transition from Web1.0 to Web2.0, and the ways we access information have dramatically changed—from relying on traditional media to accessing massive amounts of information anytime and anywhere via the internet, social media, and personal media platforms [50,51]. Particularly on social media and personal media platforms, we can find questions similar to our own and the corresponding responses [6,50,51]. However, the accuracy and comprehensiveness of information obtained in this manner can be uncertain [51]. Online responses vary greatly in quality, lacking systematic organization and authority, and the response time and outcomes of further inquiries are unpredictable. Studies have shown that answers from ChatGPT 3.5 are not only more comprehensive and empathetic than those from certified physicians on Reddit forums but, despite demonstrating high quality in assessing dementia care issues, they fall slightly short in predicting potential future problems [52,53]. When comparing responses from ChatGPT 4.0, 3.5, and those on Reddit, ChatGPT 4.0’s responses significantly surpassed the others, reaching a new level of excellence [54]. In responding to patient inquiries, LLMs also perform more accurately than Google searches and are easier to read [27]. However, they also share a common drawback: the use of LLMs in medical consultations is best accompanied by professional medical personnel to “clarify” the responses [31]. Therefore, LLMs are not suitable for independently handling any part of the diagnostic or treatment process within the medical system, but they are better suited as tools to enhance the efficiency of professional medical personnel or as mediums for personalized patient communication and education [55,56].

As technology continues to advance, hospitals are consistently innovating in all aspects of clinical diagnosis and treatment to enhance diagnostic accuracy, treatment outcomes, and patient satisfaction, representing an unstoppable trend in health care innovation [57,58]. However, balancing standardized processes with personalized patient needs often presents a challenge [59]. LLMs present an opportunity to potentially maintain standardized quality in their responses while also accommodating personalized requests. LLMs, encompassing both free and paid versions, are generally accessible to the public as open platforms [60]. Although current research does not support its use in guiding clinical decisions [61], using ChatGPT in doctor-patient communication benefits both doctors and patients [7]. Doctors can interpret and supplement ChatGPT’s responses based on their clinical experience, offering more personalized consultations to patients [31]. In addition, patients reduce their need to search for information on the internet, and their trust in physicians may be enhanced with the objective evidence provided by AI. Under the joint oversight of doctors and patients, the advantages of artificial intelligence can be fully used [62]. Nevertheless, the widespread adoption and application of LLMs still face technical and policy limitations. Technical limitations include differences in handling inputs in various languages [63], performance discrepancies between proprietary and open-source models [64], and the occurrence of “hallucinations” when faced with biased questions [65]. Since commonly used LLMs like GPT, Gemini, and Claude are proprietary, and these models are trained with significantly more data than open-source models, we can only continue to explore ways to avoid “hallucinations” instead of fixing the root cause of such issues [66,67]. In addition, policy restrictions cannot be ignored [68]. Health systems and hospitals need to develop detailed policies to regulate the clinical auxiliary use of LLMs, including ensuring patient informed consent, standardized user training, and the preservation of usage records [7]. Sound policies are essential to ensure the appropriate and efficient use of tools [65,68]. Through these measures, the safety of LLM applications in the medical field can be effectively enhanced, protecting patient rights while improving the efficiency and quality of doctor-patient communication [47,69].

### Limitations

This study has several limitations. First, both the linguistic input and the analyzed responses were in Chinese. On one hand, this choice was made to facilitate assessments by Chinese-speaking clinical experts and patients during follow-ups. On the other hand, input in different languages could introduce potential errors and biases. Second, this research only explores the feasibility of using LLMs to generate content related to SCR for patient education. The variability in surgical procedures and specialties could pose distinct challenges in patient education, which means the conclusions drawn from this study cannot be simply generalized to other disciplines. Finally, during the “Prompts Development” phase, it was found that without additional background information, SCRs are prone to be misidentified by LLMs as bridge suture repairs of the supraspinatus muscle. However, since all 3 models used were proprietary, we opted for a “Background+ Question” approach to mitigate this systematic error, without being able to investigate the reasons behind such occurrences.

### Conclusions

Claude-3-Opus, GPT-4-Turbo, and Gemini-1.5-Pro effectively addressed patient queries and generated readable presurgical education materials. However, they lacked citations and failed to explore alternative treatments, benefits, and potential risks of forgoing SCR surgery. While these LLMs can serve as valuable aids for physicians, they should not be used as standalone tools for patient education without expert oversight to ensure comprehensive and accurate information is provided.

## Supplementary material

10.2196/70047 Multimedia Appendix 1 All Questions and Answers for Claude-3-Opus, GPT-4-Turbo, and Gemini-1.5-Pro (Use GPT-4-Turbo for Chinese to English translation).

10.2196/70047 Multimedia Appendix 2 Table S1: Consistent evaluation of Fleiss kappa among raters.

10.2196/70047 Multimedia Appendix 3 Comaprison of readability by py-readability-metrics.

## Abbreviations

LLM: large language model
PEMAT: Patient Education Materials Assessment Tool
SCR: superior capsular reconstruction
