# Supplementary material for: Evaluating Large Language Models for Preoperative Patient Education in Superior Capsular Reconstruction: Comparative Study of Claude, GPT, and Gemini
Source: JMIR Perioper Med. 2025 Jun 12;8:e70047. doi: 10.2196/70047 (PMC12178570; doi:10.2196/70047)
Supplement: Multimedia Appendix 1 [file periop-v8-e70047-s001.docx]

**Supplemental File 1**

**All Questions and Answers for Claude-3-Opus, GPT-4-Turbo, and Gemini-1.5-Pro (Use GPT-4-Turbo for Chinese to English translation)**

**Claude Q1:**

**Background: The patient has been diagnosed with a massive rotator cuff tear involving supraspinatus injury, and the doctor plans to perform superior capsular reconstruction of the shoulder joint.**

**The imaging report indicates that I have a supraspinatus injury. What structure is the supraspinatus, and what causes can lead to this type of injury?**

# Supraspinatus

The supraspinatus is one of the four muscles surrounding the shoulder joint, collectively known as the rotator cuff.

It originates from the upper part of the scapula and inserts into the greater tubercle of the humerus.

## Main functions of the supraspinatus

1. Abduction of the shoulder joint, especially during the initial 0-15° of movement.

2. Assists the deltoid muscle in abducting the upper arm beyond 90°.

3. Compresses the humeral head, preventing upward displacement and maintaining shoulder joint stability.

## Common causes of supraspinatus injury

1. Degenerative changes: With age, muscle tissue gradually deteriorates, blood supply decreases, making it more susceptible to injury.

2. Repetitive shoulder activities: Frequent overhead movements (such as swimming, tennis serves, painting) can lead to muscle fatigue and injury.

3. Trauma: Falling and supporting oneself with the hand, or sudden forceful pulling of objects can cause acute injury.

4. Subacromial impingement: Due to bone spurs or abnormal acromion shape, the supraspinatus is compressed when moving under the acromion, leading to long-term wear and worsening injury.

5. Local ischemia: The rotator cuff muscles have relatively poor blood supply, especially in older adults, making them prone to ischemic injury.

Early supraspinatus injury often presents as mild pain, gradually progressing to limited shoulder joint mobility and weakness.

As the injury worsens, severe tears may occur, requiring surgical repair.

Therefore, early detection and treatment are crucial for prognosis.

Protecting the shoulder joint, avoiding overuse, and appropriate functional exercises can effectively prevent and control the progression of the condition.

**Claude Q2:**

**Background: The patient has been diagnosed with a massive rotator cuff tear involving the supraspinatus muscle, and the doctor plans to perform a superior capsular reconstruction of the shoulder joint. What is superior capsular reconstruction of the shoulder joint, what is the therapeutic principle of the surgery, and what are the indications for the surgery?**

# Superior Capsular Reconstruction is a surgical technique for treating large rotator cuff tears. Its main principles and indications are as follows:

## Surgical Principles:

1. **Repair of torn rotator cuff tendons**: The torn or retracted tendon flaps are mobilized and sutured to the anatomical position on the bone without tension, restoring the integrity and function of the rotator cuff tendons.

2. **Reconstruction of the superior capsule**: Using autologous tissue (such as long head of biceps tendon autograft) or allograft to reconstruct the superior capsule and posterior bundle, enhancing joint stability.

3. **Greater tuberosity decompression if necessary**: In some cases, an enlarged greater tuberosity may obstruct rotator cuff repair, requiring partial bone removal for decompression.

4. **Functional rehabilitation**: Early passive movement post-surgery, gradually increasing active range of motion, strengthening muscle training, and restoring shoulder joint function.

## Surgical Indications:

1. Patients over 55 years old with acute or recurrent massive rotator cuff tears (tear diameter >5cm, or involving 2 or more tendons).

2. Patients with extensive rotator cuff tears that are difficult to repair directly and those with humeral head necrosis.

3. Patients with repeated repair failures, poor tendon quality, and inability to undergo conventional rotator cuff reconstruction.

4. Elderly patients with no significant improvement after 6 months of conservative treatment, night pain affecting sleep, limited shoulder movement, and difficulty in daily activities.

5. Patients with overall health condition suitable for surgery, without severe visceral or cardiovascular complications.

**In summary, superior capsular reconstruction restores shoulder joint stability and motor function by repairing the rotator cuff and reconstructing the joint capsule. It is an advanced surgical approach for treating massive rotator cuff injuries in the elderly, but requires strict adherence to indications and active post-operative rehabilitation.**

**Claude Q3:**

**Background: The patient has been diagnosed with a massive rotator cuff tear involving the supraspinatus muscle, and the doctor plans to perform a superior capsular reconstruction of the shoulder joint. What are the common patch materials used in superior capsular reconstruction of the shoulder joint, and what are the differences between these patches?**

# The main patch materials commonly used in superior capsular reconstruction of the shoulder joint are as follows:

## 1. Autologous tissue grafts:

- Autologous tendon grafts: Taken from the patient's own tendon tissue, such as semitendinosus tendon, gracilis tendon, etc.

- Advantages: Good biocompatibility, strong integration ability. Disadvantages: May affect body function, limited extraction.

## 2. Allogeneic tissue grafts (allogeneic tendons, skin, dermal matrix, etc.):

- Advantages: No donor site issues, relatively abundant source. Disadvantages: Some risk of immune rejection and transmission of infectious diseases.

## 3. Xenogeneic biological grafts (such as porcine small intestinal submucosa matrix derivatives):

- Advantages: Abundant source, relatively economical. Disadvantages: Risk of immunogenicity and immune disease transmission.

## 4. Synthetic material grafts:

- Polyester mesh grafts: Good strength and toughness.

- Polytetrafluoroethylene (PTFE) grafts: High tensile strength, good inertness.

# Differences between various grafts:

1. Biocompatibility: Autologous tissue > Other types of allogeneic > Xenogeneic biological materials > Synthetic materials > Hybrid materials

2. Immunogenicity: Autologous tissue has no immunogenicity, while other types of materials have varying degrees of immune rejection risk.

3. Integration ability: Autologous tissue cells are more likely to survive, more conducive to tissue reconstruction, and have the strongest integration ability.

4. Strength: Synthetic materials have the highest strength, followed by autologous and allogeneic grafts, while xenogeneic materials may be relatively weaker.

5. Source and cost-effectiveness: Autologous tissue has limited sources and may damage the donor site, while allogeneic materials are more abundant.

**In conclusion, each type of graft has its advantages and disadvantages. Surgeons need to choose the best and most suitable graft material based on factors such as the patient's age, severity of the lesion, and condition of their own tissue. For massive rotator cuff tears, it may be necessary to choose stronger allogeneic tendons combined with synthetic materials, supplemented by surgical reinforcement techniques, to achieve better functional recovery.**

**Claude Q4:**

**Background: The patient has been diagnosed with a massive rotator cuff tear involving the supraspinatus muscle, and the doctor plans to perform superior capsular reconstruction of the shoulder joint. Besides the patch, does the superior capsular reconstruction surgery require the use of anchors, and do these anchors need to be removed in a second surgery?**

# In joint capsule reconstruction surgery for the shoulder joint, to securely fix the patch to the surface of the scapula and humerus, options include screws, anchors, and bone pins. The choice of these fixation devices depends on the specific circumstances of the surgeon.

## 1. Absorbable fixation devices:

- **Material:** Absorbable polymers, such as polylactic acid (PLA), polyglycolic acid (PGA), etc.

- **Advantages:** Can be absorbed by the body after a certain period, no need for a second surgery to remove.

- **Disadvantages:** Strength gradually decreases before complete absorption.

## 2. Non-absorbable fixation devices:

- **Material:** Medical-grade metals, such as titanium alloy, stainless steel, etc.

- **Advantages:** High strength, able to maintain fixation for a long time.

- **Disadvantages:** May require a second surgery for removal to avoid complications due to long-term wear.

# Factors to consider regarding the need for a second surgery to remove fixation devices:

1. **Type of fixation device:** Absorbable fixation devices usually do not need to be removed, while non-absorbable fixation devices may need removal.

2. **Patient's age and health condition:** Younger patients or those at risk of long-term wear complications may need non-absorbable fixation devices removed.

3. **Postoperative recovery:** If the patient experiences significant pain or limited function, and the fixation device does not cause a noticeable foreign body reaction, removal may not be necessary.

**In general, surgeons will communicate thoroughly with patients before surgery and decide whether to use fixation devices that require a second surgery for removal based on the patient's specific situation and surgical plan. In many cases, to avoid the risks and costs of a second surgery, doctors will prioritize absorbable fixation devices. However, for certain areas requiring high strength, such as patients with very severe rotator cuff tears, non-absorbable metal fixation devices may still be necessary to ensure long-term stability.**

**Claude Q5:**

**Background: The patient has been diagnosed with a massive rotator cuff tear involving the supraspinatus muscle, and the doctor plans to perform superior capsular reconstruction of the shoulder joint.**

**What are the surgical complications of superior capsular reconstruction of the shoulder joint?**

# Complications of Superior Capsular Reconstruction of the Shoulder Joint

**Superior capsular reconstruction of the shoulder joint is an effective surgical method for treating large rotator cuff tears, but like all surgeries, it also has some complications. The main complications include:**

1. **Infection:** Surgical site infection is one of the common complications. It may require antibiotic treatment, and in severe cases, debridement.

2. **Joint stiffness:** Postoperative limitation of joint mobility can be improved through rehabilitation, but some patients may have permanent functional impairment.

3. **Neurovascular injury:** Surgical manipulation may damage blood vessels or nerves such as the axillary nerve and musculocutaneous nerve, resulting in numbness and other symptoms.

4. **Anchor loosening:** Anchors used in reconstruction may loosen or dislodge, affecting the repair effect. Severe cases may require a second surgery.

5. **Re-tear:** Due to poor tissue healing, excessive loading, etc., the repaired tendon may re-tear, losing its stabilizing function.

6. **Pain:** Postoperative pain is common, possibly due to soft tissue damage, hardware irritation, etc., affecting the rehabilitation process. Most cases can be controlled, but some are difficult to treat.

7. **Bleeding:** Postoperative bleeding. Prolonged immobilization may lead to venous thrombosis, with a risk of pulmonary embolism in severe cases.

8. **Poor wound healing:** Diabetic patients are prone to wound healing problems, affecting aesthetics and increasing the risk of complications.

9. **Subacromial impingement:** After capsular reconstruction, the subacromial space narrows, causing impingement during movement and resulting in pain.

**In conclusion, although capsular reconstruction is effective, it still has complications. Understanding the risks and benefits is important. Patients need to weigh the pros and cons and follow medical advice for perioperative management to reduce occurrence. For elderly patients with multiple comorbidities and poor self-repair ability, more caution should be exercised in choosing this procedure.**

**Claude Q6:**

**Background: The patient is diagnosed with a massive rotator cuff tear involving injury to the supraspinatus muscle. The doctor plans to perform a superior capsular reconstruction of the shoulder joint.**

**How long is the typical rehabilitation period following superior capsular reconstruction surgery of the shoulder joint?**

# Rehabilitation time after superior capsular reconstruction surgery varies from person to person, typically taking 6-12 months. The following is a general rehabilitation timeline:

## 1. 1-4 weeks post-surgery:

- Immediately begin wearing a shoulder sling to restrict arm movement and protect the repaired tissue

- Perform finger, wrist, and elbow exercises to prevent joint stiffness

- Use ice therapy and medication as appropriate to control pain and inflammation

## 2. 6 weeks to 3 months post-surgery:

- Begin passive range of motion exercises, assisted by a physical therapist

- Gradually increase active range of motion exercises, such as scapular and prone abduction movements

- Perform scapular stability training to strengthen the muscles around the shoulder blade

## 3. 3-6 months post-surgery:

- Continue to increase active shoulder range of motion and strength training

- Begin light resistance training, such as exercises with elastic bands

- Practice activities of daily living, such as combing hair and dressing

## 4. 6-12 months post-surgery:

- Further enhance shoulder strength and endurance, restoring muscle volume

- Engage in functional training related to occupational needs or sports activities

- Achieve 80% or more function compared to pre-surgery or the opposite shoulder, allowing for return to daily activities

**In summary, full recovery takes 6-12 months, depending on the severity of the tear, quality of repair, and patient compliance. Following a standardized rehabilitation program under the guidance of a doctor and physical therapist helps accelerate recovery and achieve optimal post-surgical results.**

**Claude Q7:**

**Background: The patient has been diagnosed with a massive rotator cuff tear involving the supraspinatus muscle, and the doctor plans to perform superior capsular reconstruction of the shoulder joint.**

**What circumstances can lead to poor postoperative recovery or failure of superior capsular reconstruction surgery?**

# Superior capsular reconstruction is an effective surgical method for treating large rotator cuff tears, but in some cases it may lead to poor postoperative recovery or surgical failure. The main reasons include:

1. **Advanced age of the patient, poor healing ability.** Elderly patients (>65 years) have decreased tissue healing and blood supply reconstruction ability, resulting in slower postoperative recovery.

2. **Tear is too large or excessively complex and severe.** For example, when the tendon and periarticular soft tissues are extensively involved, tissue management becomes challenging, and even after repair, it's difficult to restore function.

3. **Muscle atrophy and fatty infiltration.** Long-term chronic shoulder and arm pain can lead to gradual replacement of muscle tissue with fatty tissue, which lacks muscle function.

4. **Concomitant shoulder pathologies.** Elderly women are prone to osteoporosis, making the tendon-bone interface fragile, which may cause internal fixation loosening and delayed healing.

5. **Infection.** Improper aseptic technique during surgery or patient immunodeficiency may lead to postoperative incision infection or even systemic infection.

6. **Acromial impingement and adhesive capsulitis.** Abnormal acromial morphology or soft tissue thickening can more easily cause soft tissue impingement during postoperative movement, leading to pain.

7. **Improper postoperative rehabilitation.** Premature weight-bearing, excessive activity, or prolonged immobilization are all detrimental to joint function recovery. Functional training should be carried out according to medical advice.

8. **History of medical conditions.** Patients with systemic diseases such as diabetes or rheumatoid arthritis may experience delayed repair healing and increased risk of complications.

*In conclusion, patient selection and precise surgical technique are crucial. Preoperatively, high-risk factors should be addressed and managed. Intraoperatively, thorough removal of lesions and minimizing damage to surrounding tissues are essential. Postoperatively, systematic rehabilitation and infection prevention are important. When necessary, coordinated treatment between internal medicine and surgery can improve surgical success rates and patient prognosis.*

**Claude Q8:**

**Background: The patient has been diagnosed with a massive rotator cuff tear involving injury to the supraspinatus muscle. The doctor plans to perform superior capsular reconstruction of the shoulder joint.**

**In superior capsular reconstruction surgery of the shoulder joint, if an autologous graft is chosen, what are the potential impacts and risks associated with harvesting tissue from the donor site?**

# Considerations and Risks When Choosing Autologous Grafts for Capsular Reconstruction in Shoulder Joint Surgery:

1. **Evaluation of donor site function for autologous tissue**:

- Common donor sites for autologous tissue grafts include the iliotibial band (ITB), quadriceps tendon, and semitendinosus tendon.

- The process of harvesting these autologous tissues can cause some trauma and postoperative pain at the donor site.

- Temporary reduction in tendon strength is a common risk.

2. **Risk of local complications at the donor site**:

- Harvesting from sites such as the iliotibial band or quadriceps tendon may lead to risks of local complications like hematoma and infection, affecting wound healing.

- Although the probability is low, vigilance is still required.

3. **Mechanical strength of the donor site**:

- Transferring autologous tendon tissue for superior capsular reconstruction affects the integrity of the donor site tendon, potentially leading to local muscle weakness.

- While long-term recovery is possible through bodily compensation, there may be some impact on lower limb function in the short term.

4. **Potential need for future autologous tissue surgery**:

- If future surgeries are required at the donor site (e.g., knee surgery), the options may be limited.

5. **Limitations in strength and quantity of autologous tissue**:

- The strength and plasticity of autologous tissue may not fully meet the mechanical strength required for reconstruction, and the tissue quantity is limited. It may be insufficient if the tear area is too large.

6. **Unclear long-term effects**:

- Currently, there is a lack of research data on the long-term effects of autologous tissue harvesting on the human body, requiring further clinical follow-up and observation.

## In summary

While using autologous tissue for superior capsular reconstruction of the shoulder joint is an effective biological material, there are indeed certain limitations and risks. In clinical application, it is necessary to weigh the pros and cons carefully based on the patient's specific condition and the doctor's experience to make an individualized treatment choice. At the same time, it is important to inform patients of the risks before surgery, strengthen rehabilitation exercises, and promote functional recovery.

**Claude Q9:**

**Background: The patient is diagnosed with a massive rotator cuff tear involving the supraspinatus muscle, and the doctor plans to perform superior capsular reconstruction of the shoulder joint.**

**What are the billable items during the superior capsular reconstruction surgery of the shoulder joint, and what surgical consumables are needed?**

# Superior Capsular Reconstruction (SCR) is a complex surgery for treating large rotator cuff tears. This surgery typically involves the following billable items and surgical materials:

## 1. Preoperative examination fees:

* Routine preoperative tests such as blood tests, ECG, chest X-ray

* MRI or CT scans if necessary

## 2. Surgical fees:

* Operating room usage fee

* Anesthesia fee (general anesthesia)

* Surgeon's fee

## 3. Hospitalization costs:

* Hospital bed charges

* Nursing care fees

* Medication costs (antibiotics, pain relievers, etc.)

## 4. Surgical materials:

* Arthroscopy system (camera, light source, monitor, etc.)

* Arthroscopic instruments (forceps, scissors, suction devices, etc.)

* Sutures (absorbable or non-absorbable)

* Anchors (for fixing rotator cuff tissue)

* High-permeability cannulas

* Drainage tubes

* Sterile surgical dressings, gowns, gloves, etc.

## 5. Postoperative rehabilitation costs:

* Rehabilitation therapy (physical therapy, functional training, etc.)

* Follow-up visits and consultation fees

Overall, Superior Capsular Reconstruction is a technically demanding surgery that requires various consumables, resulting in relatively high total costs. Specific expenses may vary depending on the patient's condition, hospital pricing, and insurance coverage. It is advisable for patients to communicate thoroughly with the hospital before surgery to understand the detailed list of charges and cost estimates, and to plan their medical expenses accordingly based on their individual circumstances.

**Claude Q10:**

**Background: The patient has been diagnosed with a massive rotator cuff tear involving the supraspinatus muscle. The doctor plans to perform a superior capsular reconstruction of the shoulder joint.**

**If the superior capsular reconstruction surgery is successful, how long will the implanted graft last, and what are the differences between various types of grafts?**

# For large rotator cuff tears with supraspinatus injury, superior capsular reconstruction with patch implantation can be expected to achieve good long-term outcomes, but the specific duration of use depends on several factors:

1. **Patch material type**:

- Common patch materials include autologous tissue (e.g., tendons, fascia), allogeneic tissue, and synthetic materials.

- Autologous and allogeneic tissues typically have higher durability than synthetic materials, but availability and complication risks are factors to consider.

2. **Patient age and activity level**:

- Younger, more active patients may subject the patch to greater mechanical stress, potentially shortening its lifespan.

- Elderly patients or those with low activity levels may have implants that last longer.

3. **Postoperative rehabilitation**:

- Good rehabilitation training helps improve joint function, reduce abnormal stress on the implant, and extend its lifespan.

- Conversely, poor rehabilitation can lead to problems and accelerate implant wear.

4. **Primary etiology**:

- If the rotator cuff tear is caused by systemic diseases such as rheumatoid arthritis, disease progression may affect long-term implant survival even after successful surgery.

## According to literature reports:

- Superior capsular reconstruction using allograft or reconstruction materials shows patch integrity rates of over 80% in 2-5 year follow-ups.

- Using autologous tendon grafts, implant integrity rates are also above 70% in long-term follow-ups of over 5 years.

## For synthetic patch materials:

- Non-absorbable materials such as polytetrafluoroethylene and polypropylene patches have high strength and good tissue compatibility, with reported cases of over 10 years of use.

- Absorbable materials like polylactic acid patches and fibrinogen matrix combined with autologous bone marrow are mainly used to provide mechanical support and induce tissue regeneration for capsule repair in the first 1-2 years post-surgery, after which they are gradually absorbed.

# In summary:

- Patches used in superior capsular reconstruction can be expected to have a clinical lifespan of 5-10 years or more.

- However, for specific long-term efficacy, choosing the right patch is only the first step; standardized surgical techniques and individualized rehabilitation plans are essential.

- For each patient, the most suitable plan should be selected based on their individual conditions and needs to achieve the best long-term clinical benefits.

**Claude Q11:**

**Background: The patient has been diagnosed with a massive rotator cuff tear involving the supraspinatus muscle, and the doctor plans to perform superior capsular reconstruction of the shoulder joint. What type of anesthesia is required for superior capsular reconstruction, how long does the surgery take, and how long is the hospital stay?**

# Information on Superior Capsular Reconstruction of the Shoulder Joint

Based on the information you provided and the patient's medical data, the patient will undergo superior capsular reconstruction of the shoulder joint to repair a large rotator cuff tear involving the supraspinatus muscle. The following information pertains to the anesthesia method, surgery duration, and hospital stay:

## 1. Anesthesia Method

Superior capsular reconstruction is typically performed under general anesthesia. General anesthesia allows the patient to remain sedated and pain-free during the procedure, facilitating the surgeon's work. The anesthesiologist will assess the patient's physical condition and surgical safety. In some cases, nerve block anesthesia may also be used in combination to better control postoperative pain.

## 2. Surgery Duration

Superior capsular reconstruction is a relatively complex procedure, and the duration of surgery depends on the severity of the tear and the surgeon's experience. Typically, the surgery takes about 2-3 hours. If the condition is more complicated or if other issues need to be addressed simultaneously, the surgery may take longer.

## 3. Hospital Stay

After superior capsular reconstruction, patients usually need to stay in the hospital for 1-2 days for close postoperative observation and rehabilitation treatment. During this time, medical staff will closely monitor your vital signs, manage pain, and guide functional exercises. Most patients can continue rehabilitation treatment at home after discharge, with regular follow-ups to assess recovery progress.

**Note:** The specific hospital stay duration and postoperative rehabilitation plan may vary. The surgical approach and rehabilitation plan need to be tailored to the individual patient's circumstances. The doctor will comprehensively evaluate factors such as the patient's age, health condition, and severity of injury to determine the most appropriate treatment plan.

**Claude document generation request:**

**Background: The patient has been diagnosed with a massive rotator cuff tear with supraspinatus injury, and the doctor plans to perform a superior capsular reconstruction of the shoulder joint. Please help me generate a systematic educational document about superior capsular reconstruction of the shoulder joint. This educational document is to be given to the patient to read during the preoperative informed consent process.**

# Dear Patient,

Based on your examination results, you have been diagnosed with a massive rotator cuff tear involving the supraspinatus muscle. To restore shoulder joint function and alleviate pain, we recommend you undergo Superior Capsular Reconstruction (SCR). This is a relatively new surgical technique specifically designed to repair large rotator cuff tears that cannot be directly sutured.

The following is a detailed explanation of the SCR procedure for your information and consideration:

## I. Surgical Objectives

1. Reconstruct the superior capsule of the shoulder joint to restore shoulder stability.

2. Prevent upward migration of the humeral head, reducing shoulder pain.

3. Improve shoulder joint mobility and enhance quality of life.

## II. Surgical Method

1. Performed under general anesthesia, typically using arthroscopic minimally invasive techniques.

2. Autologous tissue (such as gracilis tendon) or allograft tissue (such as Achilles tendon) is used as the graft ligament.

3. One end of the graft ligament is fixed to the scapula, and the other end is fixed to the greater tuberosity of the humerus.

4. Reconstruct the superior capsule stability structure to support shoulder joint function.

5. If necessary, other rotator cuff injuries will be repaired simultaneously.

## III. Surgical Risks

Like all surgeries, SCR carries certain risks, including:

1. Infection at the surgical site

2. Graft loosening or tearing

3. Shoulder stiffness

4. Vascular or nerve damage (rare)

5. Anesthesia-related complications

## IV. Postoperative Rehabilitation

1. A shoulder sling should be worn for 4-6 weeks after surgery to immobilize the injured limb.

2. Joint range of motion exercises, including passive and active movement exercises, begin after wound healing.

3. Gradually progress to functional training exercises, generally requiring 3-6 months.

4. Full recovery for daily activities may take over 6 months, while returning to heavy physical labor may take about 1 year.

## V. Expected Outcomes

After systematic rehabilitation treatment, most patients can achieve satisfactory results:

1. Significant reduction in shoulder joint pain

2. Improved shoulder joint stability and range of motion

3. Enhanced upper limb function and improved quality of life

We recommend that you fully understand the purpose, process, risks, and expected outcomes of the surgery, communicate with your family, and then make a decision. If you have any other questions, please feel free to contact us, and we will provide detailed answers.

If you decide to proceed with the surgery, please cooperate with the preoperative preparations and sign the informed consent form. We are committed to providing you with high-quality and attentive medical care.

Wishing you a speedy recovery!

XXX Hospital

Shoulder and Elbow Surgery Department

August 14, 2024

**GPT Q1:**

**Background: The patient has been diagnosed with a massive rotator cuff tear involving supraspinatus injury, and the doctor plans to perform superior capsular reconstruction of the shoulder joint.**

**The imaging report indicates that I have a supraspinatus injury. What structure is the supraspinatus, and what causes can lead to this type of injury?**

# Supraspinatus Muscle

* One of the shoulder muscles, part of the Rotator Cuff

* The Rotator Cuff consists of four muscles: supraspinatus, infraspinatus, teres minor, and subscapularis

* Located in the supraspinous fossa of the scapula

* Originates from the supraspinous fossa of the scapula, passes over the shoulder joint, and finally attaches to the greater tubercle of the humerus

## Main Functions

* Responsible for abduction and external rotation of the shoulder joint

* Plays a crucial role especially in the abduction of the shoulder joint from 30 to 90 degrees

* Helps stabilize the shoulder joint, preventing upward sliding of the humeral head in the glenoid cavity

## Common Factors of Supraspinatus Injury

1. Overuse

2. Acute injury

3. Aging

4. Shoulder joint instability

5. Subacromial impingement

## Treatment Methods

* Conservative treatment

* Medication

* Surgical treatment (in severe cases)

- For example: Superior capsular reconstruction of the shoulder joint

## Recovery Recommendations

* Perform appropriate shoulder exercises and rehabilitation training

* Develop a treatment plan in conjunction with a doctor

* Seek help from a professional physical therapist

**GPT Q2:**

**Background: The patient has been diagnosed with a massive rotator cuff tear involving the supraspinatus muscle, and the doctor plans to perform superior capsular reconstruction of the shoulder joint. What is superior capsular reconstruction of the shoulder joint, what is the therapeutic principle of the surgery, and what are the indications for the surgery?**

# Superior Capsular Reconstruction (SCR)

SCR is a relatively novel treatment method used for severe rotator cuff tears, especially when traditional repair surgery is not feasible or expected to have poor outcomes.

This surgery involves using artificial materials or autologous tissue to reconstruct the superior capsule of the shoulder joint, aiming to stabilize the shoulder joint, reduce pain, and improve function.

## Treatment Principles:

1. **Increase Stability**: By reconstructing the superior capsule of the shoulder joint, joint stability can be increased, preventing abnormal movement or dislocation of the shoulder joint.

2. **Reduce Friction**: The reconstructed capsule can reduce friction between internal structures of the shoulder joint, protecting the joint surface and extending the joint's lifespan.

3. **Improve Shoulder Function**: By stabilizing the shoulder joint, the patient's range of motion and strength may improve, enhancing daily living abilities.

## Surgical Indications:

Superior capsular reconstruction is mainly indicated in the following situations:

- **Large-scale Rotator Cuff Tears**: Especially when the rotator cuff tear cannot be repaired through suturing, or when repair results are poor.

- **Severe Joint Function Decline**: When the patient's shoulder joint function is limited, affecting daily life.

- **Intractable Pain**: To alleviate long-term pain when other non-surgical treatments (such as physical therapy, medication, etc.) are ineffective.

- **Shoulder Joint Instability**: When the shoulder joint becomes unstable due to tears, affecting normal joint movement.

Superior capsular reconstruction is a relatively new treatment technique suitable for patients with severe rotator cuff injuries where traditional treatment methods are ineffective.

This surgery can effectively restore shoulder joint stability and function, improving the patient's quality of life.

Before deciding on this surgery, please discuss the potential benefits and risks in detail with your doctor to make the best treatment plan.

**GPT Q3:**

**Background: The patient has been diagnosed with a massive rotator cuff tear involving the supraspinatus muscle, and the doctor plans to perform superior capsular reconstruction of the shoulder joint.**

**What are the commonly used patch materials in superior capsular reconstruction of the shoulder joint, and what are the differences between these patches?**

# Superior Capsular Reconstruction (SCR) and Graft Selection

**Superior Capsular Reconstruction (SCR) is a relatively new surgical technique for treating massive rotator cuff tears, aimed at improving shoulder stability and function when the rotator cuff cannot be repaired. This procedure typically involves using a graft to reconstruct the superior capsule of the shoulder joint, helping to reduce superior migration of the humeral head, thereby alleviating pain and improving shoulder function.**

## Types of Grafts Used:

### 1. Synthetic Grafts:

- These grafts are typically made from polymeric materials such as polyester, polyetheretherketone (PEEK), or other synthetic fibers.

- Advantages include availability and customization, with adjustable size and shape.

- Disadvantages may include lower compatibility with human tissue, and potentially inferior long-term stability and biomechanical performance compared to biological grafts.

### 2. Allograft (Tissue from Human Donors):

- Commonly used are processed human cadaveric or animal skin, tendons, or tissues.

- Advantages include good biocompatibility and biomechanical properties.

- Disadvantages may include potential immune rejection, risk of disease transmission, and limited supply.

### 3. Autograft (Patient's Own Tissue):

- Common autologous tissues include the fascia lata tendon.

- Advantages include perfect compatibility with no immune rejection issues.

- Disadvantages include greater surgical trauma and longer recovery time.

## Differences Between Grafts:

- **Biocompatibility**: Autografts have the best biocompatibility, followed by allografts, while synthetic grafts may have some rejection issues.

- **Biomechanical Performance**: Autografts and allografts typically better mimic the mechanical properties of the original human tissue, while synthetic grafts may be inferior in strength.

- **Infection Risk**: Autografts have the lowest risk of infection, allografts have some risk despite sterilization, and synthetic grafts generally have the lowest infection risk.

- **Surgical Complexity and Recovery Time**: Using autografts usually results in more complex surgery and longer recovery time due to the need to harvest tissue from another part of the body. Synthetic grafts and allografts can simplify this process.

**When choosing the appropriate graft type, doctors consider the patient's specific condition, surgical goals, graft availability, and patient preferences. The selection of each graft type needs to be tailored to the patient's specific situation and expected surgical outcomes.**

**GPT Q4:**

**Background: The patient has been diagnosed with a massive rotator cuff tear involving injury to the supraspinatus muscle. The doctor plans to perform superior capsular reconstruction of the shoulder joint. Besides the patch, does the superior capsular reconstruction surgery require the use of anchors, and do these anchors need to be removed in a second surgery?**

# During shoulder joint superior capsule reconstruction (also known as rotator cuff repair surgery), special small fixators such as suture anchors are typically used to secure the displaced rotator cuff tendon to the bone of the shoulder joint.

## These suture anchors are usually very small and can be made of metal or absorbable materials.

## Purpose of anchors:

Anchors are used to secure torn tendons to the bone to help the tendon heal. These suture anchors typically need to hold the tendon in place with sutures as healing occurs.

## Whether a second surgery is needed for removal:

1. **Metal anchors:** This type of anchor usually does not need to be removed through a second surgery, as they are designed to remain in the body permanently. Unless the anchor causes inflammation, displacement, or reacts with surrounding tissue, it is typically not removed.

2. **Absorbable anchors:** These anchors are made of special materials that can gradually dissolve or be absorbed and broken down in the body. Therefore, this type of anchor also does not require removal through a second surgery.

## Other considerations:

* **Postoperative recovery:** Recovery after surgery includes gradual functional therapy to help improve the function and stability of the shoulder joint.

* **Potential risks:** Any surgery can carry risks of complications, such as infection or anchor displacement, but these can usually be addressed through follow-up treatment.

## In summary, anchors used in rotator cuff repair surgery generally do not need to be removed through a second surgery; they are designed to remain in the body long-term.

## Postoperative management mainly focuses on appropriate rehabilitation and monitoring for anchor-related complications.

## Before surgery, your doctor will choose the most suitable type of anchor based on your specific situation and will explain the surgical process and postoperative rehabilitation plan in detail.

**GPT Q5:**

**Background: The patient has been diagnosed with a massive rotator cuff tear involving the supraspinatus muscle, and the doctor plans to perform a superior capsular reconstruction of the shoulder joint.**

**What are the surgical complications of superior capsular reconstruction of the shoulder joint?**

# Superior Capsular Reconstruction (SCR)

**Superior Capsular Reconstruction (SCR) is a surgical procedure used to treat irreparable rotator cuff tears. This surgery reconstructs the superior capsule of the shoulder joint using a graft, thereby increasing the stability and function of the shoulder joint. Although SCR surgery can significantly improve a patient's pain and functional status, like all surgeries, it may also come with some complications. Common complications include:**

1. **Infection**: The surgical area may become infected, which may require antibiotic treatment, and in severe cases, additional surgery.

2. **Nerve damage**: Nerves around the shoulder may be damaged during the surgery, leading to abnormal sensation or weakened motor function in the shoulder.

3. **Graft failure**: The graft may shift, loosen, or rupture due to inadequate fixation, overuse, or biomechanical factors. In such cases, revision surgery may be necessary.

4. **Intraoperative or postoperative bleeding**: A hematoma may form in the surgical area after the operation. Severe bleeding may require additional surgery.

5. **Shoulder stiffness**: Shoulder stiffness or limited range of motion may occur after surgery, usually requiring physical therapy to improve.

6. **Pain**: There may be local pain for some time after surgery, which usually improves over time with appropriate rehabilitation training.

7. **Recurrent shoulder instability**: Although SCR aims to provide shoulder stability, in some cases, the shoulder joint may still have instability issues.

8. **Fracture**: Tools or techniques used during surgery may cause fractures in nearby bones (such as the humerus or scapula).

9. **DVT/PE (Deep Vein Thrombosis/Pulmonary Embolism)**: Although less common, prolonged surgery and reduced activity during the recovery period can increase the risk of blood clot formation.

**Understanding these potential complications can help patients and doctors better assess the benefits and potential risks of the surgery, and take appropriate preventive and management measures to reduce risks. Before undergoing SCR surgery, it is very important to communicate in detail with the doctor about these risks and the patient's specific health conditions.**

**GPT Q6:**

**Background: The patient has been diagnosed with a massive rotator cuff tear involving injury to the supraspinatus muscle. The doctor plans to perform a superior capsular reconstruction of the shoulder joint.**

**How long is the typical rehabilitation period following superior capsular reconstruction surgery of the shoulder joint?**

# Superior Capsular Reconstruction (SCR)

SCR is a relatively new surgical procedure aimed at treating severe rotator cuff tears that have not responded to conservative treatment, especially in cases where the rotator cuff is irreparable. This surgery typically uses artificial ligaments or autologous tissue to reconstruct the superior capsule of the shoulder joint, thereby improving shoulder stability and function.

## Overview of Recovery Time

Recovery time after Superior Capsular Reconstruction can vary from several months to a year, depending on various factors such as surgical details, patient age, health condition, and dedication to the rehabilitation process. Here's a general recovery timeline:

### 1. Early Post-operative Period (0-6 weeks):

- The goal is to protect the surgical site and reduce inflammation.

- Patients typically need to wear a brace to control shoulder movement.

- Gentle passive movements may be performed to promote blood circulation and prevent joint stiffness.

### 2. Mid-term Rehabilitation (6-12 weeks):

- Gradually increase the range of passive movements.

- Begin active muscle movements.

- Light strength training using elastic bands may be introduced.

### 3. Late Rehabilitation (after 12 weeks):

- Increase shoulder range of motion.

- Strengthen shoulder muscles.

- Begin more challenging functional exercise movements, such as lifting and rotation.

### 4. Full Recovery:

- Complete recovery may take 6 months to 1 year.

- Some patients may require more time to return to pre-surgery activity levels.

## Key Factors in Rehabilitation

- **Professional Guidance**: Following a physical therapist's instructions for rehabilitation training is crucial to ensure correct recovery steps and gradual progression of exercise intensity.

- **Personal Effort**: The patient's active cooperation and daily persistence in exercises are also vital for recovery.

- **Following Medical Advice**: Strictly adhere to the doctor's recommendations for rehabilitation activities, and do not increase the load prematurely.

## Summary

Recovery after SCR is a long-term process that requires continuous effort from the patient. Each person's recovery is unique, so it's very important to work closely with your medical team, regularly assess recovery progress, and adjust the rehabilitation plan accordingly.

**GPT Q7:**

**Background: The patient is diagnosed with a massive rotator cuff tear involving the supraspinatus muscle, and the doctor plans to perform superior capsular reconstruction of the shoulder joint.**

**What circumstances can lead to poor postoperative recovery or failure of superior capsular reconstruction surgery?**

# Superior Capsular Reconstruction of the Shoulder Joint

Superior capsular reconstruction of the shoulder joint is a surgical procedure used to treat severe rotator cuff tears, especially when the rotator cuff cannot be repaired through suturing. In this surgery, doctors reconstruct the superior capsule of the shoulder joint to improve shoulder stability and function. The success of the surgery depends not only on the procedure itself but is also limited by various factors, mainly including:

## 1. Preoperative Factors:

- **Patient age and overall health**: Older patients or those with other health issues (such as diabetes, heart disease, etc.) have poorer healing abilities.

- **Muscle and tendon quality**: Poor quality of shoulder muscles and tendons may lead to insufficient healing ability of repaired tissue after surgery.

## 2. Surgical Technique:

- **Technical execution**: Improper surgical technique, such as insecure fixation or incorrect positioning of capsular reconstruction material, may lead to displacement failure.

- **Choice of reconstruction material**: The material used may be incompatible with the patient's internal environment or of poor quality, affecting surgical outcomes.

## 3. Postoperative Management:

- **Rehabilitation process**: Patients not following an appropriate rehabilitation plan after surgery, such as engaging in excessive activity too early, may lead to damage to the newly repaired capsule.

- **Physical therapy**: Inappropriate physical therapy methods may result in poor capsule repair or inflammation.

## 4. Infection and Inflammation:

- **Surgical infection**: Postoperative infection can severely affect the healing process and may lead to surgical failure.

- **Immune response**: Patients may develop an immune response to the reconstruction material, leading to chronic inflammation or rejection.

## 5. Individual Patient Factors:

- **Compliance**: Patient non-compliance with medical advice, such as smoking, drinking alcohol, or not taking medications as prescribed, can affect healing.

To improve the success rate of surgery, doctors and patients need to work together, including selecting appropriate surgical techniques, ensuring proper postoperative management, and patients following professional rehabilitation guidance. Continuous doctor-patient communication before surgery is also crucial to ensure that all patient questions are addressed fully and promptly, and open communication is maintained.

**GPT Q8:**

**Background: The patient has been diagnosed with a massive rotator cuff tear involving injury to the supraspinatus muscle. The doctor plans to perform superior capsular reconstruction of the shoulder joint for the patient.**

**In superior capsular reconstruction surgery of the shoulder joint, if an autologous graft is chosen, what are the potential impacts and risks associated with the donor site where the autologous tissue is harvested?**

# Considerations for Using Autologous Tissue in Superior Capsular Reconstruction of the Shoulder Joint

When performing superior capsular reconstruction of the shoulder joint, if autologous tissue is chosen as a graft, common donor sites include the fascia lata of the thigh, hamstring tendon, and latissimus dorsi tendon. Using autologous tissue offers better biocompatibility but also comes with some risks and issues:

## Impact and Risks of Autologous Donor Sites:

1. **Trauma and Pain**: The harvesting surgery itself causes additional trauma and pain. For example, harvesting from the fascia lata of the thigh may result in pain, swelling, and scarring in the donor area.

2. **Infection Risk**: Any surgery carries a risk of infection, and the harvesting procedure increases the overall risk of infection for the surgery.

3. **Functional Impairment**: Harvesting may cause functional impairment of the tissue at the donor site. For instance, using a tendon may affect the function of related muscle strength.

4. **Increased Healing Time**: Both surgical sites (donor site and reconstruction site) require more time to heal, which may prolong the overall recovery time of the surgery.

5. **Aesthetic Concerns**: The donor site may leave scars, potentially affecting the patient's appearance.

6. **Blood Circulation Impact**: The harvesting surgery may affect local blood circulation, especially when using larger areas of tissue or if the area's tissue is already relatively thin.

## Clinical Decision Factors:

- **Patient's Health Status**: Consider the patient's age, overall health condition, and other medical issues to assess their ability to withstand two surgical sites.

- **Surgical Experience and Equipment**: The surgeon's experience and available surgical techniques are also important factors in determining the use of autologous tissue.

- **Patient Understanding and Needs**: Thorough communication with the patient is necessary to understand their expectations for surgical outcomes and tolerance for recovery time, which helps in deciding the treatment plan.

Before deciding to use an autologous tissue graft, doctors will comprehensively consider the above factors and have detailed discussions with patients to ensure the most suitable treatment plan is chosen. In some cases, the use of allograft tissue or synthetic materials may be considered as alternatives to reduce surgical risks and recovery time.

**GPT Q9:**

**Background: The patient has been diagnosed with a massive rotator cuff tear involving the supraspinatus muscle, and the doctor plans to perform superior capsular reconstruction of the shoulder joint.**

**What are the billable items during the superior capsular reconstruction surgery of the shoulder joint, and what surgical consumables are needed?**

# Superior Capsular Reconstruction (SCR)

## Overview

* A surgical procedure used to treat severe rotator cuff tears

* Particularly suitable for cases where the rotator cuff cannot be directly repaired

* Typically involves using artificial or biological materials to reconstruct the superior capsule of the shoulder joint

* Aims to help restore stability to the shoulder joint

## Billable Items

1. Surgical Fee

* Includes fees for the lead surgeon, assistants, and anesthesiologist

2. Hospital Stay Fee

* Varies based on the patient's length of stay and room type

3. Anesthesia Fee

* Varies depending on the type of anesthesia used and duration

4. Examination Fee

* Includes necessary pre- and post-operative examinations such as X-rays, MRI, CT scans, etc.

5. Medication Fee

* Medications used during and after surgery

6. Physical Therapy Fee

* Multidisciplinary physical therapy that may be required post-surgery

7. Special Materials Fee

* Cost of special consumables and implants used in surgery

## Surgical Consumables

1. Artificial Joint Capsule Material

* May use synthetic materials or biological materials from donors

2. Anchors

* Used to secure the material to the bone

3. Sutures

* Used to stitch torn muscles and secure the new joint capsule

4. Hemostatic Materials

* Used to control and prevent bleeding during the procedure

5. Surgical Instruments

* Includes knives, forceps, scissors, and specialized surgical tools

* Possible need for special instruments for arthroscopy

6. Anesthetic Drugs

* Drugs required for local or general anesthesia

## Other Considerations

* Post-operative Brace

* A special shoulder brace may be needed post-surgery to stabilize the joint

* Post-operative Medications

* Including pain relief medications and possible antibiotics to prevent infection

## Notes

* When planning the surgery, the doctor and medical team will provide a detailed list of costs and consumables based on the patient's specific situation

* Patients can use this information for consultation and budget planning

**GPT Q10:**

**Background: The patient has been diagnosed with a massive rotator cuff tear involving the supraspinatus muscle, and the doctor plans to perform superior capsular reconstruction of the shoulder joint. If the superior capsular reconstruction surgery is successful, how long will the implanted graft last, and what are the differences between various types of grafts?**

# Superior Capsular Reconstruction Surgery for Shoulder Joint

Superior capsular reconstruction surgery is a treatment for severe rotator cuff tears, especially when the rotator cuff cannot be repaired. This surgery involves implanting a graft to reconstruct the superior capsule of the shoulder joint, providing necessary stability and function. Below is an overview of the advantages and disadvantages of different types of grafts, their lifespan, and characteristics:

## 1. Types of Grafts

### Autologous Tendon

- Source: Patient's own tendons from other parts of the body.

- Advantages: Good biocompatibility, low risk of rejection.

- Disadvantages: Requires additional surgery for harvesting, may increase recovery time and pain.

- Lifespan: Theoretically can last a lifetime, but depends on healing after implantation and tendon quality.

### Allograft Tendon

- Source: Donors.

- Advantages: No need to harvest tissue from the patient, reducing surgical site pain and recovery time.

- Disadvantages: Some risk of rejection, requires long-term monitoring.

- Lifespan: Can last over 10 years in most cases, but specific longevity is affected by multiple factors.

### Artificial Synthetic Materials

- Types: Polyethylene terephthalate (PET), etc.

- Advantages: Can be mass-produced with consistent quality and size.

- Disadvantages: May have slightly lower biocompatibility with human tissue, long-term stability needs further observation.

- Lifespan: Usually expected to last 5-10 years, but requires regular check-ups until eventual degradation and loss of function.

## 2. Lifespan of Grafts

The lifespan of grafts is influenced by various factors, including the type of implant material, patient's age, activity level, and quality of post-operative rehabilitation. Autologous tendons are generally considered to have the longest lifespan as they are living tissue and more likely to integrate with the patient's own tissue. Allograft tendons and artificial synthetic materials may have shorter lifespans, depending on material properties and interaction with human tissue.

## 3. Key Factors for Success

- Preoperative preparation: Detailed assessment of the patient's condition and comprehensive preoperative planning.

- Intraoperative technique: Precise surgical technique and correct graft positioning.

- Postoperative rehabilitation: Strict adherence to rehabilitation plans and appropriate functional exercises.

In conclusion, superior capsular reconstruction surgery is an effective treatment option, with different types of grafts potentially varying in lifespan and efficacy. The choice of graft type should be based on the patient's physical condition and the doctor's recommendations.

**GPT Q11:**

**Background: The patient is diagnosed with a massive rotator cuff tear involving the supraspinatus muscle injury. The doctor plans to perform superior capsular reconstruction of the shoulder joint. What type of anesthesia is required for superior capsular reconstruction, how long does the surgery take, and how long is the hospital stay?**

# Superior Capsular Reconstruction Surgery is typically a relatively complex shoulder procedure, primarily used to repair joint capsule laxity caused by rotator cuff injuries. The following is a general introduction to the anesthesia method, surgery duration, and hospital stay:

## Anesthesia Method

- **General Anesthesia**: This is the most common anesthesia method, as it keeps the patient still and pain-free throughout the entire surgical process. General anesthesia is more conducive to controlling the patient's vital signs during surgery and ensures the procedure can be completed smoothly.

- **Local Anesthesia with Sedation**: For some specific cases, doctors may choose local anesthesia combined with mild sedation. This method can reduce the potential risks associated with general anesthesia and is particularly suitable for patients with severe systemic diseases.

## Surgery Duration

- Typically, Superior Capsular Reconstruction Surgery may take **between 1 to 3 hours**, depending on the severity of the injury and the complexity of the surgery. If there are extensive tears or other tissues that need repair, the surgery time may be extended accordingly.

## Hospital Stay

- The length of hospital stay depends on the complexity of the surgery and the patient's recovery. Generally, the hospital stay may last **2 to 5 days**. Patients need sufficient observation time after surgery to monitor the wound condition and potential complications, and to begin initial rehabilitation therapy.

- After discharge, patients usually need several weeks or even months of physical therapy to help restore shoulder function and reduce the risk of surgical complications.

## Summary

Before undergoing Superior Capsular Reconstruction Surgery, patients should discuss the details of each instruction with their doctor to determine the most suitable anesthesia plan. The doctor will develop a personalized treatment plan based on the patient's health condition and surgical needs.

**GPT document generation request:**

**Background: The patient has been diagnosed with a massive rotator cuff tear with supraspinatus injury, and the doctor plans to perform superior capsular reconstruction of the shoulder joint. Please help me generate a systematic educational document about superior capsular reconstruction of the shoulder joint. This educational document is given to patients to read during the preoperative informed consent process.**

# Superior Capsular Reconstruction (SCR) Patient Education Document

## I. Surgery Overview

**Superior Capsular Reconstruction (SCR) is a surgical procedure used to treat severe rotator cuff tears that cannot be directly repaired by suturing. This surgery reconstructs the superior capsule of the shoulder joint using artificial materials or autologous tissue to improve shoulder joint stability and function.**

## II. Surgical Objectives

**The SCR surgery aims to:**

* **Restore shoulder stability and reduce abnormal joint movement.**

* **Alleviate pain caused by rotator cuff tears.**

* **Improve shoulder range of motion and overall strength.**

## III. Indications

**Your doctor will consider SCR for massive rotator cuff tears due to supraspinatus injury where traditional surgery may not achieve ideal repair results. SCR is suitable for patients in the following conditions, especially those with capsular laxity due to rotator cuff tears:**

## IV. Surgical Procedure

1. **Anesthesia and Preparation: The surgery is performed under general anesthesia, ensuring you won't feel pain throughout the procedure.**

2. **Arthroscopic Examination: An arthroscope is inserted through small incisions to examine the shoulder joint's interior in detail.**

3. **Cleaning and Assessment: The shoulder joint is cleaned and the extent of damage is assessed.**

4. **Graft Implantation: Pre-prepared material (artificial synthetic material or donor tissue) is implanted in the superior part of the shoulder joint to reconstruct the capsule.**

5. **Fixation: The reconstructed material is fixed in place using anchors and/or sutures.**

6. **Closure: After implantation, incisions are sutured and dressed.**

## V. Post-operative Recovery

1. **Initial Phase: A shoulder brace is worn post-surgery to limit shoulder movement and prevent graft displacement.**

2. **Physical Therapy: Progressive physical therapy is conducted under medical guidance to gradually increase shoulder range of motion.**

3. **Follow-up: Regular hospital visits to monitor recovery progress and graft status.**

## VI. Risks and Complications

**Potential risks of SCR surgery include:**

* **Infection**

* **Graft displacement or failure**

* **Neurovascular injury**

* **Persistent pain or unexpected lack of functional improvement**

## VII. Conclusion

**Superior Capsular Reconstruction is an advanced treatment method that offers a therapeutic possibility for difficult-to-repair rotator cuff tears. Before undergoing surgery, it's crucial to understand the procedure's purpose, process, recovery period, and potential risks. Ensure thorough communication with your doctor to clarify all your questions.**

## VIII. Informed Consent

**Before the surgery, you need to sign an informed consent form, confirming that you fully understand all aspects of the surgery and agree to receive treatment. If you have any questions, please discuss them with your medical team before signing.**

**This document is intended to help you make an informed decision and does not constitute an absolute commitment to your upcoming surgery. We will work together with you to improve your shoulder health through this surgery.**

**Gemini Q1:**

**Background: The patient has been diagnosed with a massive rotator cuff tear involving the supraspinatus muscle injury. The doctor plans to perform superior capsular reconstruction of the shoulder joint. The imaging report indicates supraspinatus injury. What is the structure of the supraspinatus, and what causes can lead to this type of injury?**

# Supraspinatus Muscle

The supraspinatus muscle is located in the shoulder and is one of the four muscles that make up the rotator cuff. It is responsible for shoulder abduction (lifting the arm away from the body) and rotation.

## Causes of Supraspinatus Injury

There are many causes of supraspinatus injury, including:

- **Trauma**: For example, falling directly on the shoulder can cause a supraspinatus tear.

- **Overuse**: Repetitive overhead activities, such as baseball, tennis, and volleyball, can put stress on the supraspinatus and lead to tears over time.

- **Shoulder Impingement Syndrome**: The acromion is part of the shoulder blade that rubs or "impinges" on the supraspinatus and the subacromial bursa (a fluid-filled sac between the supraspinatus tendon and the acromion) when the arm is raised. This friction can lead to tendon inflammation (tendinitis) and tears.

- **Degenerative Changes**: As we age, the supraspinatus tendon gradually wears down and becomes more susceptible to tears. Other factors, such as poor blood supply, can also lead to tendon degeneration.

## Symptoms of Supraspinatus Injury

The symptoms of a supraspinatus injury depend on the severity of the damage and may include:

- **Pain**: Especially when lifting the arm or performing overhead activities.

- **Weakness**: Difficulty lifting or rotating the arm.

- **Limited Range of Motion**: Reduced shoulder mobility.

- **Stiffness**: The shoulder feels stiff or "catches" during movement.

**If you suspect you may have a supraspinatus injury, be sure to consult a doctor for diagnosis and treatment options.**

**Gemini Q2:**

**Background: The patient is diagnosed with a massive rotator cuff tear involving the supraspinatus muscle injury. The doctor plans to perform superior capsular reconstruction (SCR) of the shoulder joint. What is superior capsular reconstruction of the shoulder joint, what is the therapeutic principle of the surgery, and what are the indications for the surgery?**

# Superior Capsular Reconstruction Surgery

## Superior Capsular Reconstruction (SCR) is an emerging technique for treating massive irreparable rotator cuff tears.

## This surgery restores the mechanical balance of the shoulder joint by reconstructing the stabilizing structure above the shoulder joint, thereby reducing pain and improving function.

### Surgical Treatment Principle

- Rotator cuff injuries, especially supraspinatus injuries, lead to loss of active support above the shoulder joint, causing upward displacement of the humeral head and disrupting the normal biomechanical relationship of the glenohumeral joint.

- This results in pain, weakness, limited mobility, and even joint degeneration.

- SCR surgery reconstructs the stabilizing structure above the shoulder joint by implanting a graft (usually autologous fascia lata or allograft tendon).

- The graft is attached to the glenoid fossa and coracoid process of the scapula, mimicking the function of the supraspinatus, pulling the humeral head downward and restoring normal alignment and stability of the glenohumeral joint.

### Surgical Indications

SCR surgery is mainly applicable in the following situations:

- Massive irreparable rotator cuff tears: For patients with extensive rotator cuff tears, poor muscle quality, and inability to perform direct suture repair, SCR surgery provides an alternative treatment option.

- Failed rotator cuff repair: For patients who have undergone rotator cuff repair surgery with unsatisfactory results, SCR surgery can serve as a salvage procedure.

- Shoulder pain and functional impairment: For patients with shoulder pain and functional impairment unresponsive to conservative treatment, if imaging studies show rotator cuff tear and humeral head displacement, SCR surgery may be considered.

## It should be noted that SCR surgery is not suitable for all patients with rotator cuff injuries.

## The indications for surgery need to be assessed by a professional orthopedic surgeon based on the patient's specific condition.

### Summary

- SCR surgery is an effective method for treating massive irreparable rotator cuff tears, capable of relieving pain and improving function.

- However, the indications for surgery need to be strictly controlled, and assessment and operation by professional physicians are required.

**Gemini Q3:**

**Background: The patient is diagnosed with a massive rotator cuff tear involving the supraspinatus muscle, and the doctor plans to perform superior capsular reconstruction of the shoulder joint. What are the commonly used patch materials in superior capsular reconstruction of the shoulder joint, and what are the differences between these patches?**

# Patches commonly used in superior capsular reconstruction of the shoulder joint are mainly divided into two categories: autografts and allografts.

## 1. Autografts

### Advantages:

* Good biocompatibility, no rejection reactions.

* Strong tissue healing ability.

* Relatively low cost.

### Disadvantages:

* Requires harvesting from other parts of the patient's body, increasing surgery time and trauma.

* Potential complications at the donor site, such as pain, infection, nerve damage, etc.

* Limited available tissue for harvesting, especially for thinner patients.

### Commonly used autografts:

* Tensor fascia lata: The most commonly used autograft, with moderate thickness and strength, relatively easy to harvest.

* Semitendinosus/gracilis tendon: High strength, but larger harvesting volume.

## 2. Allografts

### Advantages:

* No need for autologous harvesting, reducing surgery time and trauma.

* Abundant material sources, not limited by the patient's own conditions.

* Some products are specially processed, with high tensile strength and good biomechanical properties.

### Disadvantages:

* Risk of rejection reactions, although the incidence has been greatly reduced with modern processing techniques.

* Relatively higher cost.

### Commonly used allografts:

* Decellularized dermal matrix: Derived from human or animal dermal tissue, processed with special techniques to remove cellular components while retaining the natural collagen structure, with good biocompatibility and tissue compatibility.

* Synthetic materials: Such as polyester, polypropylene, etc., with certain strength and toughness, but relatively poor biocompatibility.

## Differences between patches:

* Source: Autografts come from the patient's own body, while allografts come from other human or animal tissues or are synthetically produced.

* Biocompatibility: Autografts have the best biocompatibility, followed by decellularized dermal matrix, with synthetic materials being relatively poor.

* Mechanical properties: Different material patches vary in strength, elasticity, and tensile mechanical properties, requiring selection based on the patient's specific situation.

* Cost: Autografts are relatively low-cost, while allografts are more expensive.

## Choosing the appropriate patch:

Selecting the appropriate patch requires consideration of factors such as the patient's age, gender, physical condition, activity requirements, degree of tear, and the doctor's experience. It is recommended to communicate fully with your doctor, weigh the pros and cons, and choose the treatment plan that best suits you.

**Gemini Q4:**

**Background: The patient has been diagnosed with a massive rotator cuff tear involving the supraspinatus muscle. The doctor plans to perform superior capsular reconstruction of the shoulder joint. Besides the patch, does the superior capsular reconstruction surgery require the use of anchors, and if so, do these anchors need to be removed in a second surgery?**

# Superior Capsular Reconstruction of the Shoulder Joint

Superior capsular reconstruction of the shoulder joint typically requires the use of suture anchors or bone grafts (patches) fixed to the scapula. These anchors are usually made of absorbable materials, such as polylactic acid, but may also be made of metal or polyether ether ketone (PEEK).

Whether a second surgery is needed to remove the anchors depends on the material of the anchors used:

* Absorbable anchors: These anchors are naturally absorbed by the body within one to two years after surgery, so no second surgery is required for removal.

* Metal or PEEK anchors: These anchors are usually permanent and do not need to be removed unless they cause discomfort or complications.

It's important to note that:

* Not all superior capsular reconstructions of the shoulder joint require the use of anchors. In some cases, sutures can be used to fix the graft to the scapula.

* Whether anchors are needed and what type of anchors to use depends on the specific circumstances of the patient, such as the size and location of the tear, the patient's age and activity level, and the surgeon's personal preference.

It is recommended that you discuss the surgical plan with your doctor, including whether anchors are needed and what type of anchors to use. The doctor will choose the most suitable treatment plan for you based on your specific situation.

**Gemini Q5:**

**Background: The patient has been diagnosed with a massive rotator cuff tear involving the supraspinatus muscle, and the doctor plans to perform superior capsular reconstruction of the shoulder joint. What are the surgical complications of superior capsular reconstruction of the shoulder joint?**

# Potential Complications of Superior Capsular Reconstruction Surgery for the Shoulder Joint

While superior capsular reconstruction surgery for the shoulder joint is generally safe, like any surgery, it carries potential risks of complications. These complications can be categorized as follows:

## 1. Anesthesia-related complications:

- Any surgery may involve anesthesia-related risks, such as allergic reactions to anesthetic drugs, breathing difficulties, and cardiac arrhythmias.

## 2. Surgery-related complications:

- **Infection**: There is a risk of infection with any surgery, and shoulder surgery is no exception.

- **Bleeding**: Bleeding may occur during or after the surgery.

- **Nerve damage**: There are many nerves around the shoulder joint that could be damaged during surgery, leading to numbness, weakness, or pain in the shoulder or arm.

- **Joint stiffness**: Joint stiffness may occur after surgery, affecting the range of motion of the shoulder joint.

- **Screw or anchor problems**: Screws or anchors used in the surgery may loosen, shift, or break.

- **Rotator cuff re-tear**: Even after successful surgery, the rotator cuff may tear again, especially under strenuous activities.

## 3. Other complications:

- **Persistent pain**: Some patients may experience persistent pain after surgery.

- **Rehabilitation difficulties**: Superior capsular reconstruction surgery requires long-term rehabilitation training, and some patients may encounter difficulties with rehabilitation.

- **Unsatisfactory surgical outcomes**: Not all patients benefit from surgery; some patients' symptoms may not improve or may even worsen.

It's important to note that the occurrence rate of these complications is relatively low, and most patients successfully complete the surgery and recover well. The surgeon's experience and skill level, the patient's age and health condition, and post-operative care all affect the incidence of complications.

If you are planning to undergo superior capsular reconstruction surgery, be sure to discuss the risks and benefits of the surgery, as well as the post-operative rehabilitation plan, in detail with your doctor.

**Gemini Q6:**

**Background: The patient has been diagnosed with a massive rotator cuff tear involving injury to the supraspinatus muscle. The doctor plans to perform a superior capsular reconstruction of the shoulder joint. How long is the typical rehabilitation period following superior capsular reconstruction surgery of the shoulder joint?**

# For patients with massive rotator cuff tears following supraspinatus injury who undergo superior capsular reconstruction of the shoulder joint, the rehabilitation time depends on several factors, including the patient's age, physical condition, extent of the tear, surgical method, and post-operative recovery speed.

## Generally, complete recovery takes 3-6 months, or even longer.

### Here is an approximate timeline of the rehabilitation process:

#### 0-6 weeks post-surgery:

* The main goals of this phase are to control inflammation, alleviate pain, and protect the repaired capsule.

* Patients need to wear a shoulder sling and avoid excessive shoulder joint movement.

* Passive shoulder joint movements are performed, gradually increasing the range of motion.

* Gentle muscle contraction exercises are conducted.

#### 6-12 weeks post-surgery:

* Begin active shoulder joint movements, gradually increasing range of motion and strength.

* Perform light resistance training, such as using resistance bands.

* Some daily living activities can be resumed, such as dressing and grooming.

#### 3-6 months post-surgery:

* Continue to increase shoulder joint range of motion and strength.

* Perform more challenging strength training, such as using heavier dumbbells or equipment.

* Gradually resume some sports activities, such as swimming and cycling.

#### 6 months post-surgery and beyond:

* Most patients can return to near-normal activity levels.

* More vigorous sports can be resumed, such as running and basketball.

* Care should be taken to avoid overusing the shoulder joint to prevent re-injury.

**It's important to note that this is only a general timeline, and each patient's rehabilitation process may vary. Patients need to maintain close communication with their doctor and physical therapist, develop a reasonable rehabilitation plan based on their individual situation, and strictly follow the plan to achieve the best recovery results.**

### Here are some suggestions to aid recovery:

* Actively cooperate with the treatment plans of doctors and physical therapists.

* Take medications as prescribed to control pain and inflammation.

* Persist with rehabilitation exercises, progressing gradually and avoiding overexertion.

* Maintain good lifestyle habits, quit smoking, limit alcohol consumption, and ensure adequate sleep.

* Pay attention to changes in the shoulder joint and seek medical attention promptly if discomfort occurs.

**I hope this information is helpful to you. Please remember that this is only for reference, and you should consult your doctor or physical therapist for specific advice.**

**Gemini Q7:**

**Background: The patient is diagnosed with a massive rotator cuff tear involving the supraspinatus muscle, and the doctor plans to perform superior capsular reconstruction of the shoulder joint. Under what circumstances would the postoperative recovery be poor or the surgery fail for superior capsular reconstruction of the shoulder joint?**

# Superior Capsular Reconstruction (SCR)

## Overview

SCR is an emerging and highly effective surgical technique for patients with massive irreparable rotator cuff tears accompanied by superior migration of the humeral head. Although this technique has made significant progress, there are still factors that can lead to suboptimal surgical outcomes.

## Common Causes of Poor Prognosis in Superior Capsular Reconstruction Surgery

### Surgery-related Factors

- **Inappropriate graft selection and fixation**:

- The key to SCR surgery lies in selecting a suitable graft and ensuring secure fixation.

- If the graft size is inappropriate, biomechanical properties are poor, or fixation is not secure enough, it may lead to graft loosening, displacement, and affect surgical outcomes.

- **Intraoperative injury**:

- During surgery, surrounding blood vessels, nerves, or other soft tissues may be damaged, leading to postoperative complications such as pain and functional impairment.

- **Laxity in capsular reconstruction**:

- If the reconstructed capsule is too loose to effectively limit the upward migration of the humeral head, it will lead to surgical failure.

- **Inappropriate postoperative rehabilitation plan**:

- SCR requires a strict rehabilitation plan to restore joint range of motion, strength, and stability.

- If the rehabilitation plan is unreasonable, such as engaging in strenuous activities too early or insufficient rehabilitation exercises, it may lead to graft damage, pain, joint stiffness, and other issues affecting surgical outcomes.

### Patient-related Factors

- **Age and health status**:

- Advanced age, diabetes, osteoporosis, rheumatoid arthritis, and other underlying conditions, as well as poor lifestyle habits such as smoking, can affect tissue healing ability and surgical outcomes.

- **Severity of shoulder joint pathology**:

- If the patient's rotator cuff tear is too extensive, muscle fatty infiltration is severe, or shoulder joint degeneration is advanced, it may be difficult to fully restore shoulder function even after SCR surgery.

- **Unrealistic preoperative functional expectations**:

- The main goals of SCR surgery are to reduce pain and improve function, but it cannot fully restore the shoulder to its pre-injury state.

- If patients have unrealistic expectations for surgical outcomes, they may feel disappointed postoperatively.

- **Poor compliance**:

- Patients need to actively cooperate with doctors in rehabilitation training and follow medication instructions after surgery.

- If patient compliance is poor, failing to perform rehabilitation exercises on time or self-medicating, it will affect surgical outcomes.

### Other Factors

- **Infection**:

- Any surgery carries a risk of infection, and SCR is no exception.

- Infection can cause symptoms such as pain, swelling, and fever, and in severe cases may require reoperation for debridement.

- **Nerve injury**:

- During SCR surgery, surrounding nerves may be damaged, leading to symptoms such as shoulder numbness and weakness.

- **Joint stiffness**:

- If the shoulder joint is immobilized for a long time after surgery or rehabilitation exercises are limited, it may lead to joint stiffness, affecting shoulder joint range of motion.

## Conclusion

Superior Capsular Reconstruction is a complex surgery, and its success depends on multiple factors. To improve the success rate of the surgery, doctors need to comprehensively assess the patient's condition, develop individualized treatment plans, and ensure proper postoperative management. Patients also need to actively cooperate with the doctor's treatment and maintain good lifestyle habits to achieve the best treatment outcomes.

**Gemini Q8:**

**Background: The patient is diagnosed with a massive rotator cuff tear involving injury to the supraspinatus muscle. The doctor plans to perform superior capsular reconstruction of the shoulder joint. In superior capsular reconstruction surgery, if an autologous graft is chosen, what impacts and risks are associated with the site from which the autologous tissue is harvested?**

# In shoulder joint superior capsule reconstruction surgery, if autologous grafts are chosen, commonly used autologous tissue sites include:

* Tensor fascia lata tendon: This is the most commonly used source of autologous grafts.

* Impact: After harvesting the tensor fascia lata tendon, there may be weakness in leg external rotation or slight decrease in strength, as well as pain, numbness, or sensory abnormalities at the harvest site.

* Risks: Infection at the harvest site, hematoma formation, nerve damage, etc.

* Gracilis tendon: Also a commonly used source of autologous grafts.

* Impact: After harvesting the gracilis tendon, there may be a slight decrease in medial knee support strength, as well as pain, numbness, or sensory abnormalities at the harvest site.

* Risks: Infection at the harvest site, hematoma formation, nerve damage, etc.

**Overall, the complication rate at the tendon harvest site for autologous grafts is low, and most patients recover well after surgery. However, choosing an autologous graft requires weighing the pros and cons and fully communicating with the doctor to select the most suitable treatment plan.**

## Here are some factors to consider:

* Patient age and physical condition: Younger, healthier patients are more likely to tolerate autologous tissue transplant surgery.

* Severity and location of the tear: For massive rotator cuff tears, autologous grafts may provide more ideal support.

* Patient's expected activity level: For people engaged in activities requiring extensive shoulder joint movement, autologous grafts may better meet their functional needs.

**The final treatment plan should be decided jointly by the doctor and patient based on the patient's specific circumstances.**

**Gemini Q9:**

**Background: The patient has been diagnosed with a massive rotator cuff tear involving injury to the supraspinatus muscle. The doctor plans to perform a superior capsular reconstruction of the shoulder joint. What are the billable items during the superior capsular reconstruction surgery process, and what surgical consumables are needed?**

# Shoulder Joint Superior Capsule Reconstruction Surgery Involves Multiple Surgical Steps and Consumables

The following lists the main items and consumables, but specific items and usage may vary depending on factors such as region, hospital level, and patient condition.

## I. Surgical Tools

* Surgical instruments: Including operating table, anesthesia, and common instruments used in operating rooms.

* Anesthesia: Calculated based on the method of anesthesia (general anesthesia or brachial plexus block) and duration.

* Disposable consumables: Single-use items used during surgery, such as surgical gowns, gloves, gauze, dressings, etc.

## II. Material Costs

* Arthroscopy system consumables: Such as arthroscopes, shavers, radiofrequency electrodes, irrigation systems, etc.

* Repair materials:

* Suture anchors: Implants used to reattach torn supraspinatus tendons to the humeral head. Prices vary greatly depending on material and specifications; absorbable or non-absorbable materials can be chosen.

* High-strength sutures: Used for suturing tendons and joint capsules, typically non-absorbable sutures are used.

## III. Other Expenses

* Examination fees: Preoperative examinations, such as X-rays, MRIs, etc.

* Laboratory fees: Preoperative tests, such as blood tests, coagulation function tests, etc.

* Medication costs: Postoperative medications for anti-inflammation, pain relief, swelling reduction, etc.

* Bed fees: Costs for hospital bed during the inpatient stay.

* Nursing fees: Costs for nursing care during the hospital stay.

The following lists some commonly used surgical consumable brands for reference:

* Arthroscopy systems: Stryker, Smith & Nephew, Johnson & Johnson, etc.

* Suture anchors: Stryker, Smith & Nephew, Zimmer, Medtronic, etc.

* High-strength sutures: Ethicon, Smith & Nephew, etc.

It should be noted that:

* The above only covers items and consumables involved in general situations; specific circumstances should be discussed with the surgeon or relevant departments.

* Some hospitals may offer package pricing based on actual conditions; specific pricing standards are subject to hospital announcements.

* Patients can choose different consumables and treatment plans based on their individual circumstances; doctors will provide advice based on the patient's specific situation.

Hope this information is helpful to you!

**Gemini Q10:**

**Background: The patient has been diagnosed with a massive rotator cuff tear involving injury to the supraspinatus muscle. The doctor plans to perform superior capsular reconstruction (SCR) of the shoulder joint. If the superior capsular reconstruction surgery is successful, how long will the implanted graft last, and what are the differences between various types of grafts?**

# Superior Capsular Reconstruction (SCR) is an emerging surgical technique for treating massive irreparable rotator cuff tears, aimed at restoring shoulder stability and function by reconstructing the superior capsule.

## While this technique is promising, the long-term effects of using different types of grafts are yet to be fully understood.

### Graft Longevity

#### Currently, there is no exact data on the longevity of grafts after Superior Capsular Reconstruction.

#### Some studies suggest that grafts can last for several years or even longer without needing replacement.

#### Longer-term research data is needed on their durability.

#### Factors affecting graft longevity include:

- Patient factors: age, activity level, health conditions (e.g., diabetes)

- Surgical factors: graft type, fixation method, surgical technique

- Post-operative rehabilitation: adherence to care plan, avoiding strenuous activities

### Different Types of Grafts

#### The main types of grafts used for Superior Capsular Reconstruction fall into two categories:

1. Autografts: Tendons taken from other parts of the patient's own body, such as the fascia lata or gracilis tendon.

- Advantages: Good biocompatibility, strong integration, theoretically lower risk of infection.

- Disadvantages: Requires additional surgical incision to harvest the tendon, potentially increasing surgery time and risk of complications.

2. Allografts: Tendons taken from donors, processed and sterilized.

- Advantages: No additional surgical incision required, shorter surgery time.

- Disadvantages: Risk of immune rejection and disease transmission, although the risk is very low.

### Differences

#### Currently, there is insufficient evidence to prove which type of graft has a clear advantage in Superior Capsular Reconstruction.

#### Some studies suggest that autografts may have better biomechanical properties and long-term effects, but more research is needed to confirm this.

### Summary

#### Superior Capsular Reconstruction is a new surgical technique for treating massive irreparable rotator cuff tears, and the type and longevity of the implanted graft are important concerns for patients and doctors.

#### Although there is currently no exact data on graft longevity, some studies suggest that grafts can last for several years or even longer.

#### Many factors affect graft longevity, including patient factors, surgical factors, and post-operative rehabilitation.

#### Currently, there is insufficient evidence to prove which type of graft has a clear advantage.

#### Patients should discuss with their doctors which type of graft and surgical approach is best suited for their specific situation.

**Gemini Q11:**

**Background: The patient has been diagnosed with a massive rotator cuff tear involving the supraspinatus muscle, and the doctor plans to perform superior capsular reconstruction of the shoulder joint. What type of anesthesia is required for superior capsular reconstruction, how long does the surgery take, and what is the expected hospital stay duration?**

# For patients with massive rotator cuff tears after supraspinatus injury undergoing superior capsular reconstruction of the shoulder joint, general anesthesia combined with regional block anesthesia is typically used.

## Below are detailed explanations of the anesthesia method, surgery duration, and hospital stay:

### Anesthesia method:

* **General anesthesia**: The patient is in a completely unconscious state during surgery, requiring endotracheal intubation and ventilator-assisted breathing.

* **Regional block anesthesia**: A commonly used method is brachial plexus block, which involves injecting local anesthetic around the brachial plexus to block sensory nerve conduction from the shoulder to the arm. The patient remains awake during surgery but loses sensation in the shoulder and arm.

### Surgery duration:

* The surgery time for shoulder capsular reconstruction is typically 2-3 hours, with the specific duration depending on factors such as the severity of the tear, the complexity of the surgical procedure, and individual patient differences.

### Hospital stay:

* Typically, patients need to stay in the hospital for 1-2 days. The length of stay depends on the patient's postoperative recovery, including pain control, wound healing, and ability to perform simple daily activities.

### Other important information:

* Before surgery, doctors will develop an individualized anesthesia plan based on the patient's specific circumstances, such as age, physical condition, and medical history.

* After surgery, patients need to undergo a period of functional rehabilitation training to restore shoulder joint mobility and strength.

**Please note**: The above information is for reference only. Please consult your doctor for specific details.

**Gemini document generation request:**

**Background: The patient has been diagnosed with a massive rotator cuff tear involving the supraspinatus muscle, and the doctor plans to perform superior capsular reconstruction of the shoulder joint. Please help me generate a systematic educational document about superior capsular reconstruction of the shoulder joint. This educational document is given to the patient to read during the preoperative informed consent process.**

# Superior Capsular Reconstruction of the Shoulder Joint: Surgical Informed Consent Form

This document is intended to help you understand the information related to superior capsular reconstruction of the shoulder joint, including the purpose of the surgery, steps, risks, and postoperative rehabilitation, so that you can make an informed decision about whether to accept surgical treatment.

## I. What is the rotator cuff?

The rotator cuff is composed of tendons from four muscles (supraspinatus, infraspinatus, teres minor, and subscapularis) that surround the shoulder joint. It acts like a sleeve, stabilizing the humeral head (top of the upper arm bone) in the glenoid cavity of the scapula, helping you perform various shoulder movements such as lifting, rotating, and abducting.

## II. What is a rotator cuff tear?

A rotator cuff tear refers to the tearing of one or more tendons that make up the rotator cuff. The most commonly injured tendon is the supraspinatus tendon. Rotator cuff tears can be caused by a single injury (such as a fall or direct impact) or by long-term overuse or repetitive movements.

## III. What is a massive rotator cuff tear?

A massive rotator cuff tear refers to a large extent of rotator cuff tearing, usually indicating a supraspinatus tendon tear longer than 5 cm, or a tear involving two or more rotator cuff tendons.

## IV. What are the symptoms of a rotator cuff tear?

* Shoulder joint pain, especially worsening at night or during activity.

* Shoulder weakness, difficulty lifting or rotating the arm.

* Limited shoulder mobility, such as difficulty combing hair, dressing, or reaching for objects.

* Clicking or grinding sensation in the shoulder joint.

## V. How is a rotator cuff tear diagnosed?

* Physical examination: The doctor will check your shoulder joint's range of motion, stability, muscle strength, and tenderness.

* Imaging:

* X-ray: Can rule out bone fractures and other injuries.

* Ultrasound: Can dynamically observe the morphology and movement of rotator cuff tendons.

* Magnetic Resonance Imaging (MRI): Can clearly show the extent and location of rotator cuff tears, as well as other potential injuries.

## VI. What is superior capsular reconstruction of the shoulder joint?

For massive rotator cuff tears, traditional rotator cuff repair is difficult due to the large defect. In this case, superior capsular reconstruction of the shoulder joint can be chosen. This surgery restores shoulder joint function by implanting biological materials to reconstruct the stabilizing structure above the shoulder joint.

## VII. Surgical steps:

1. Anesthesia: General anesthesia or brachial plexus block anesthesia can be chosen.

2. Incision: The doctor will make an incision on the front or back of your shoulder joint.

3. Joint cleaning: The doctor will clean inflammatory tissue and bone spurs in the joint.

4. Graft implantation: The doctor will suture and fix biological materials to the scapula and humeral head to reconstruct the stabilizing structure above the shoulder joint.

5. Suturing: Close the incision.

## VIII. Surgical risks and complications:

All surgeries have risks and complications, and superior capsular reconstruction of the shoulder joint is no exception. Common risks and complications include:

* Anesthesia risks

* Infection

* Bleeding

* Nerve damage

* Vascular injury

* Shoulder stiffness

* Graft loosening

* Surgical failure

## IX. Postoperative rehabilitation:

Postoperative rehabilitation is crucial for restoring shoulder joint function. You need to follow your doctor's guidance and undergo systematic rehabilitation training, including:

* Early passive movement: Wear a shoulder brace in the early postoperative period and perform passive movements to prevent joint stiffness.

* Active movement: Gradually begin active shoulder joint movements to restore range of motion.

* Weight-bearing training: Gradually increase weight load to restore muscle strength.

* Daily living activity training: Restore ability to perform daily activities.

## X. Other considerations:

* Before surgery: You need to inform your doctor about your medical history, allergies, and current medications.

* Before surgery: You need to fast for a certain period.

* After surgery: You will need to stay in the hospital for observation for a few days.

* After surgery: You need regular follow-ups to assess recovery progress.

## XI. Choice:

Accepting surgical treatment is an important decision. You need to weigh the pros and cons of surgery and communicate fully with your doctor to choose the most suitable treatment plan for you.

## XII. Disclaimer:

This article is for reference only and cannot replace professional medical advice. If you have any questions, please consult your doctor.

Please read the above content carefully and sign the surgical informed consent form based on your full understanding.
