# Supplementary material for: Evaluating Large Language Models for Preoperative Patient Education in Superior Capsular Reconstruction: Comparative Study of Claude, GPT, and Gemini
Source: JMIR Perioper Med. 2025 Jun 12;8:e70047. doi: 10.2196/70047 (PMC12178570; doi:10.2196/70047)
Supplement: Multimedia Appendix 2 [file periop-v8-e70047-s002.docx]

**Supplemental File 2**

Table s1 Consistent evaluation of Fleiss kappa among raters

|  | Item | [Fleiss kappa](http://www.baidu.com/link?url=8gk3WGJG5oEUITvdVcbcoAhukuEhkXPUwc0WhKhCDr9tXiqhDxRrIdMOVOvAEO_x3rrkWapWcFrwqjJ_RfVwuatgSfUQgYHFA8jXtFmzeim) | Interpretation | *P* value |
| --- | --- | --- | --- | --- |
| Claude-3-Opus | Correctness | 0.342 | Fair agreement | 0.005 |
|  | Completeness | 0.381 | Fair agreement | 0.005 |
|  | Logic | 0.533 | Moderate agreement | <0.001 |
|  | Potential harm | 0.398 | Fair agreement | 0.005 |
|  | Overall satisfaction | 0.360 | Fair agreement | <0.001 |
|  | DISCERN | 0.333 | Fair agreement | 0.013 |
|  | PEMAT-P | 0.723 | Substantial agreement | <0.001 |
| GPT-4-Turbo | Correctness | 0.641 | Substantial agreement | <0.001 |
|  | Completeness | 0.409 | Moderate agreement | 0.003 |
|  | Logic | 0.641 | Substantial agreement | <0.001 |
|  | Potential harm | 0.809 | Almost perfect agreement | <0.001 |
|  | Overall satisfaction | 0.361 | Slight agreement | <0.001 |
|  | DISCERN | 0.508 | Moderate agreement | <0.001 |
|  | PEMAT-P | 0.600 | Moderate agreement | <0.001 |
| Gemini-1.5-Pro | Correctness | 1 | Perfect agreement | - |
|  | Completeness | 0.431 | Moderate agreement | 0.013 |
|  | Logic | 1 | Perfect agreement | - |
|  | Potential harm | 1 | Perfect agreement | - |
|  | Overall satisfaction | 0.103 | Fair agreement | 0.462 |
|  | DISCERN | 0.508 | Moderate agreement | <0.001 |
|  | PEMAT-P | 1 | Perfect agreement | - |
| Patient evaluation | Readability, educational value and overall rating | 0.302 | Fair agreement | <0.001 |
| Consistent interpretation of Fleiss kappa values: Poor agreement(<0.01); Slight agreement(.01-.20); Fair agreement(.21-.40); Moderate agreement(.41-.60); Substantial agreement(.61-.80); Almost perfect agreement(.81-1.00); | | | | |
