# Supplementary material for: Evaluating Large Language Models for Preoperative Patient Education in Superior Capsular Reconstruction: Comparative Study of Claude, GPT, and Gemini
Source: JMIR Perioper Med. 2025 Jun 12;8:e70047. doi: 10.2196/70047 (PMC12178570; doi:10.2196/70047)
Supplement: Multimedia Appendix 3 [file periop-v8-e70047-s003.docx]

Multimedia Appendix 3. Comparison of readability by py-readability-metris.

| Readability metrics | Claude | Gemini | GPT | *P* |
| --- | --- | --- | --- | --- |
| a_flesch_kincaid_score (mean ±SD) | 14.41 ±2.23 | 14.71 ±1.75 | 15.12 ±1.90 | .655 |
| a_flesch_kincaid_grade_level (mean ±SD) | 14.46 ±2.18 | 14.62 ±1.85 | 15.15 ±1.99 | .658 |
| a_flesch_score (mean ±SD) | 19.06 ±8.57 | 22.22 ±10.16 | 19.14 ±7.78 | .592 |
| a_flesch_ease = very_confusing (%) | 11 (84.6) | 9 (69.2) | 10 (76.9) | .648 |
| a_flesch_grade_levels = ['college_graduate'] (%) | 11 (84.6) | 9 (69.2) | 10 (76.9) | .648 |
| a_gunning_fog_score (mean ±SD) | 16.95 ±2.79 | 17.08 ±2.50 | 17.17 ±2.01 | .975 |
| a_gunning_fog_grade_level = college_graduate (%) | 5 (38.5) | 6 (46.2) | 8 (61.5) | .488 |
| a_coleman_liau_score (mean ±SD) | 17.31 ±1.46 | 16.99 ±2.11 | 17.19 ±1.51 | .890 |
| a_coleman_liau_grade_level (mean ±SD) | 17.23 ±1.59 | 17.00 ±2.20 | 17.23 ±1.69 | .935 |
| a_dale_chall_score (mean ±SD) | 12.31 ±0.87 | 11.63 ±1.00 | 12.21 ±0.78 | .120 |
| a_dale_chall_grade_levels = ['college_graduate'] (%) | 13 (100.0) | 12 (92.3) | 13 (100.0) | .358 |
| a_linsear_write_score (mean ±SD) | 12.70 ±5.03 | 14.98 ±3.28 | 15.35 ±3.78 | .218 |
| a_linsear_write_grade_level (mean ±SD) | 12.62 ±5.12 | 14.92 ±3.35 | 15.46 ±3.80 | .194 |
| a_smog_score (mean ±SD) | 1.06 ±3.82 | 1.20 ±4.33 | 0.00 ±0.00 | .608 |
| a_smog_grade_level (mean ±SD) | 1.08 ±3.88 | 1.23 ±4.44 | 0.00 ±0.00 | .608 |
